# Ratiometric Singlet Oxygen Sensor Based on BODIPY-DPA Dyad

**DOI:** 10.3390/molecules27249060

**Published:** 2022-12-19

**Authors:** Alexey A. Pakhomov, Anastasia S. Belova, Arevik G. Khchoyan, Yuriy N. Kononevich, Dmitriy S. Ionov, Margarita A. Maksimova, Anastasiya Yu. Frolova, Mikhail V. Alfimov, Vladimir I. Martynov, Aziz M. Muzafarov

**Affiliations:** 1 M.M. Shemyakin and Yu.A. Ovchinnikov Institute of Bioorganic Chemistry, Russian Academy of Sciences, 117997 Moscow, Russia; 2 A.N. Nesmeyanov Institute of Organoelement Compounds, Russian Academy of Sciences, 119991 Moscow, Russia; 3 Faculty of Chemical-Pharmaceutical Technologies and Biomedical Preparations, Mendeleev University of Chemical Technology of Russia, 125047 Moscow, Russia; 4 Photochemistry Center, FSRC “Crystallography and Photonics”, Russian Academy of Sciences, 119421 Moscow, Russia; 5 Chemistry Department, M.V. Lomonosov Moscow State University, 119991 Moscow, Russia; 6 Moscow Institute of Physics and Technology, State University, 141707 Dolgoprudny, Russia; 7 N.S. Enikolopov Institute of Synthetic Polymeric Materials, Russian Academy of Sciences, 117393 Moscow, Russia

**Keywords:** reactive oxygen species, singlet oxygen, sensor, BODIPY, DPA, anthracene

## Abstract

Compounds sensitive to reactive oxygen species are widely used in the study of processes in living cells and in the development of therapeutic agents for photodynamic therapy. In the present work, we have synthesized a dyad in which the BODIPY dye is chemically bound to 9,10-diphenylanthracene (DPA). Here, DPA acts as a specific sensor of singlet oxygen and BODIPY as a reference dye. We studied the photophysical properties of the BODIPY-DPA dyad and showed that energy transfer occurs between the chromophores. As a result, the compound has excitation maxima in the absorption region of both DPA and BODIPY, but the fluorescence emission occurs mainly from BODIPY. In the presence of singlet oxygen, the excitation maximum of DPA decreases, while the intensity of the excitation maximum of BODIPY remains almost unchanged. This allows the BODIPY-DPA dyad to be used as a ratiometric sensor of singlet oxygen.

## 1. Introduction

**Figure 1:**
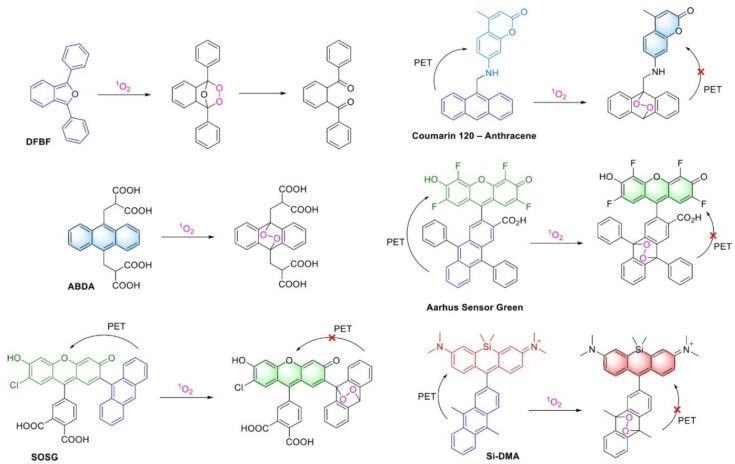
Examples of colorimetric and fluorescent singlet oxygen sensors, as well as reactions leading to changes in its optical properties.

Reactive oxygen species (ROS) play an essential role in cellular metabolism [1,2,3] as well as in photodynamic therapy (PDT) of diseases [4,5,6]. Singlet oxygen (^1^O_2_) is one of the most important ROS involved in many intracellular processes in normal conditions and in pathology, and it is also actively generated by photosensitizers in PDT [7]. A number of sensors for singlet oxygen detection have now been developed (Figure 1). Colorimetric sensors allow to monitor the ^1^O_2_ level spectrophotometrically. These sensors are based on dyes, whose absorption spectrum changes considerably after binding to singlet oxygen. In organic solvents, 1,3-diphenylisobenzofuran (DPBF) is usually used to monitor the dynamics of ^1^O_2_ generation [8], whereas in aqueous solutions, the water-soluble anthracene derivative 9,10-anthracenediyl-bis(methylene)dimalonic acid (ABDA) is usually used [9]. It was shown that DPBF can react with ^1^O_2_ with high efficiency, but also exhibits activity against other ROS [10], while anthracene derivatives demonstrate extremely high specificity to ^1^O_2_ [11]. To monitor the generation of ^1^O_2_ in living cells, fluorogenic sensors are widely used. Singlet Oxygen Sensor Green (SOSG) is commercially available and is probably the most commonly used one [12,13,14], but other sensors fluorescing in green (Aarhus Sensor Green) [15], red (Si-DMA) [16], or blue (Coumarin 120—Anthracene) [17] spectral region are also described. They consist of dyads of anthracene, responsible for ^1^O_2_ binding, and a chromophore capable of fluorescence in the corresponding spectral range. Due to photoelectron transfer (PET) between the anthracene and the chromophore, the dye initially does not fluoresce, but upon binding of ^1^O_2_ to anthracene the chromophore “lights up” (Figure 1). Such sensors are intensiometric, meaning that they enable monitoring the process by the dye fluorescence intensity in a given channel. In contrast, ratiometric sensors allow the determining of target analytes by the ratio of fluorescence signal intensity in different channels [18]. This makes it possible to determine analyte concentrations more precisely. In the case of ^1^O_2_ sensors, the ratio of intensities in different channels can be used to distinguish the termination of the ^1^O_2_ generation process from the termination of a reactive probe. Recently, ratiometric sensors of singlet oxygen based on 1,3-diarylisobenzofurans have been described [19]; however, like DPBF, they should demonstrate side specificity to other ROS.

In the present work, we have synthesized a ratiometric sensor of singlet oxygen based on a pair of 9,10-diphenylanthracene (DPA) and 4,4-difluoro-4-bora-3a,4a-diazas-indacene (BODIPY) dyes. Here, DPA acts as a sensitive module, whereas BODIPY acts as a reference dye whose fluorescence is independent of the presence of ^1^O_2_. Thus, the synthesized probe, on the one hand, is ratiometric and, on the other hand, is based on the highly specific ^1^O_2_ anthracene.

**Figure 2:**
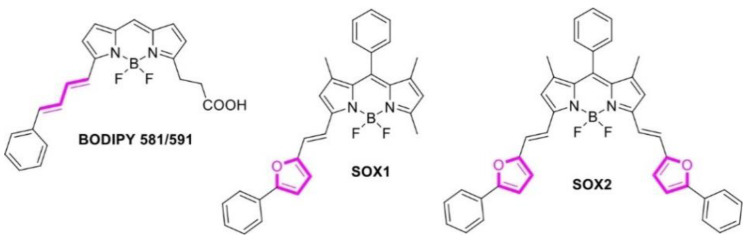
Structures of some BODIPY derivatives that can be used as sensors for ^1^O_2_. ROS binding sites are marked with magenta.

BODIPY derivatives have recently become extremely popular for imaging and sensing in living systems [20,21,22,23]. They are usually characterized by high fluorescence quantum yield, narrow excitation and emission bands of fluorescence, high photostability, and, perhaps most attractively, they can be readily derivatized to produce dyes with new optical or sensory properties [24,25,26,27]. It has been shown that BODIPY derivatives can react with and generate ROS [28,29,30,31,32]. Thus, BODIPY 581/691 can react with ^1^O_2_ through double bonds [33], and a series of SOX sensors can react through furan moieties [34] (Figure 2). However, polyenes and furan derivatives are not highly specific to ^1^O_2_ and readily react with other ROS [11,35]. Several anthracene-BODIPY conjugates have recently been reported, but they have been used as ROS generators for PDT [36,37,38,39].

## 2. Results and Discussion

### 2.1. Synthesis of BODIPY-DPA Dyad

Dyad, containing BODIPY and DPA fragments in the structure, was prepared from carboxylic derivatives of BODIPY and DPA. Carboxyl-containing 9,10-diphenylanthracene **7** (DPA-COOH) was prepared in several steps according to Scheme 1. BODIPY-COOMe **8** and BODIPY-COOH **9** were prepared as described earlier [40]. BODIPY-COOH **9** was reacted with 10-fold excess of ethylenediamine in the presence of DCC, NHS and DMAP to form amino-derivative **10**, which was reacted with DPA-COOH **7** at the same conditions to form BODIPY-DPA dyad **11**. All products were purified by column chromatography on silica. The structure of the synthesized compounds was determined by ^1^H, ^13^C, ^19^F NMR, IR and MS analysis (Supplementary Materials, Figures S1–S18).

### 2.2. Optical Properties of the Synthesized Compounds

**Figure 3:**
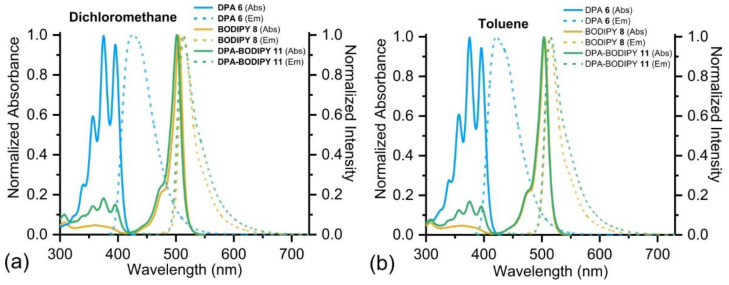
Normalized UV-visible absorption and emission spectra of DPA-COOMe **11**, BODIPY-COOMe **8** and BODIPY-DPA **6** in dichloromethane (**a**) and toluene (**b**) at room temperature. Excitation at 375 nm for DPA-COOMe **6**, BODIPY-DPA **11** and at 475 nm for BODIPY-COOMe **8**.

The optical properties of monomers DPA-COOMe **6** and BODIPY-COOMe **8**, as well as of dyad BODIPY-DPA **11,** were studied in detail in various solvents at room temperature and are presented in Table 1. Normalized electronic absorption and emission spectra of compounds **6**, **8** and **11** are shown in Figure 3 and Figures S19–S22. As seen in Figure 3, the absorption spectra of DPA-COOMe **6** in dichloromethane and toluene consist of practically identical structured bands. The first intense bands, which can be attributed to the S0→S1 transition, have several resolved peaks at about 396, 375 (maximum), 357, 340 and 324 nm. The emission spectra of DPA-COOMe **6** consist of one broad peak with maxima at approximately 425 nm in dichloromethane and 421 nm in toluene. The fluorescence quantum yields of DPA-COOMe **6** in dichloromethane and toluene are 0.83 and 0.74, respectively.

The absorption bands of BODIPY-COOMe **8** in dichloromethane and toluene, which are attributed to the BODIPY S0→S1 transition, are sharp and well-resolved, with maxima at 502 nm (in dichloromethane) and 505 nm (in toluene). Fluorescence spectra of BODIPY-COOMe **8** consist of one narrow peak with maxima at 511 nm in dichloromethane and 514 nm in toluene. Fluorescence quantum yields are 0.86 and 0.95 in dichloromethane and toluene, respectively.

The absorption spectra of BODIPY-DPA dyad **11** are close to linear combinations of the absorption spectra of the DPA and BODIPY moieties with the corresponding maxima at about 375 and 500 nm. When BODIPY-DPA **11** is excited at 375 nm, dual emission at wavelengths of about 425 nm (from DPA moiety) and 515 nm (from the BODIPY moiety) are observed. However, it is clearly seen that the fluorescence of DPA is negligible, which can be explained by energy transfer from the excited donor unit (DPA) to the acceptor (BODIPY). The quantum yields of dyad **11** (when excited at 375 nm) and the BODIPY moiety of **11** (when excited at 475 nm) are significant, but somewhat lower than for BODIPY-COOMe **8**, in both solvents (Table 1). Comparison of the shape of the fluorescence spectra of BODIPY-COOMe **8** with BODIPY-DPA **11** indicates its broadening for the dyad **11** that can be attributed to the appearance of a new band corresponding to conformers in which DPA and BODIPY moieties are in close proximity. Analogous spectral behavior was observed for the DBMBF2-BODIPY dyad described earlier [41].

Using the obtained extinction coefficients and quantum yields, we calculated the energy transfer efficiency (Φ_ET_) from donor to acceptor by two methods (Table 1). According to the method considering only the energy loss by donor, the energy transfer efficiency is 99% (Table 2, Φ_ET_^6^). However, if energy received by acceptor is taken into account the efficiency of energy transfer from the donor to the acceptor is about 70% (Table 2, Φ_ET_^7^). Thus, the additional ways of energy loss by donor, such as conformation rearrangement process should be considered. It has recently been shown that in multichromophore BODIPY compounds, chromophore interactions within the molecule can lead to the formation of different types of aggregates and the appearance of excimer-like states. This significantly affects the optical properties of the compound, including brightness [42]. Importantly, these properties strongly depend on the parameters of the medium in which the dye is located.

**Figure 4:**
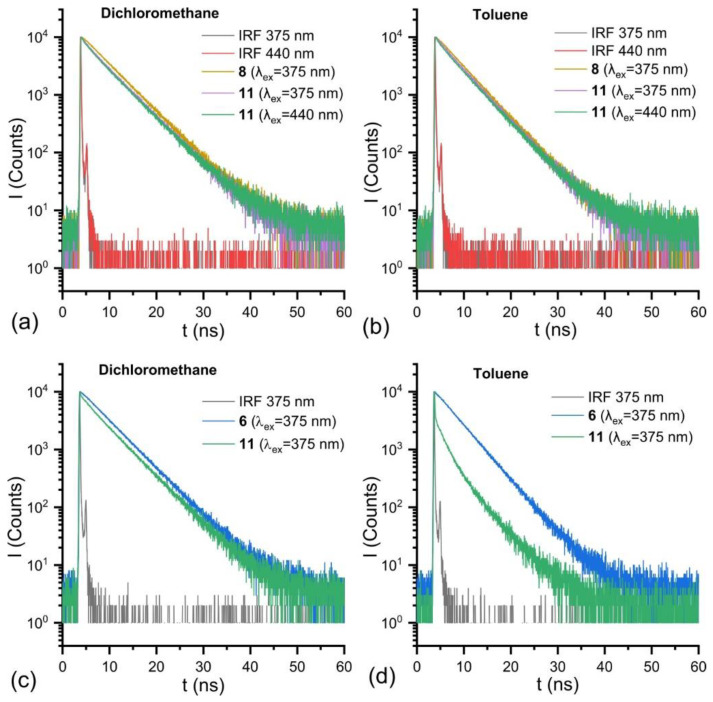
Fluorescence decay kinetics of compound **8** and **11** in dichloromethane (**a**) and toluene (**b**) obtained at 511 nm and for compounds **6** and **11** in dichloromethane (**c**) and toluene (**d**) measured at 431 nm (excitation wavelengths are written in the corresponding figures).

**Figure A1:**
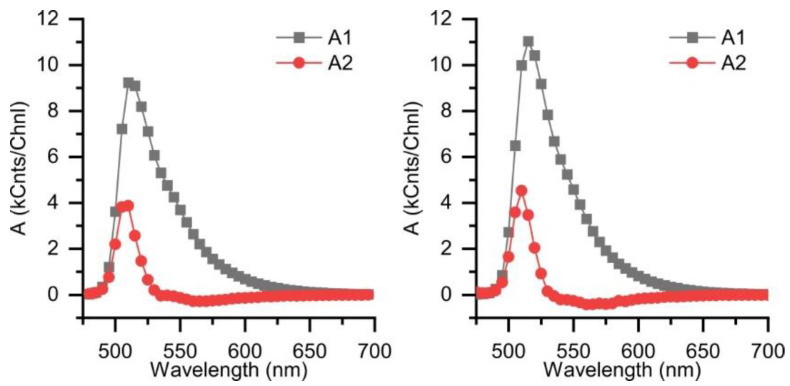
Spectra of amplitudes A_1,_ A_2_ obtained from global fitting of time-resolved fluorescence spectra of **11** in dichloromethane (**left**) and toluene (**right**) λ_ex_ = 440 nm.

**Figure A2:**
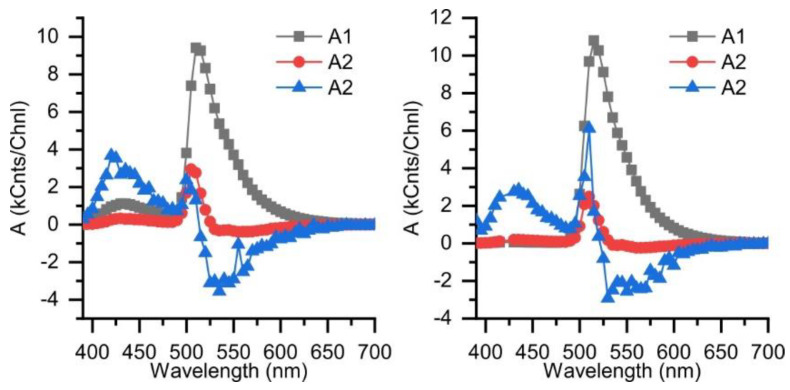
Spectra of amplitudes A_1_–A_3_ obtained from global fitting of time-resolved fluorescence spectra of **11** in dichloromethane (**left**) and toluene (**right**) λ_ex_ = 375 nm.

For a more detailed analysis of the interchromophore interactions in the DPA-BODIPY dyad, we analyzed the kinetics of fluorescence decays. The fluorescence decay kinetics of compounds **6** and **8** are monoexponential and do not depend on excitation wavelength. The lifetimes are summarized in Table 2. The fluorescence decay of compound **11** measured at 511 nm with excitation at 440 nm when only BODIPY moiety should be excited is biexponential (Figure 4a,b and Table 2). The global fitting of time-resolved fluorescence emission spectra (TRES) obtained with excitation at 440 nm in both toluene and dichloromethane demonstrates the same results (Table 2 and Appendix A, Figure A1). The spectra of amplitudes corresponding to exponential term with lifetime τ_2_ (0.63 and 0.5 ns in dichloromethane and toluene, respectively) have negative amplitudes in 530–600 nm range (Figure A1), indicating a conformational rearrangement process. The TRES obtained for solution of **11** in toluene with 375 nm excitation requires a three-exponential model for a satisfactory fit. The same model can be applied to TRES obtained in dichloromethane, but this does not lead to significant improvement in fitting quality (Table 2 and Appendix A, Figure A2). The presence of an additional fast component is clearly seen in Figure 4c,d. The additional fast component with a lifetime of about 60 ps in toluene and about 50 ps in dichloromethane appears in this case, which apparently can be attributed to energy transfer process.

Thus, the analysis of the optical properties of BODIPY-DPA **11** clearly indicates the presence of interchromophore interactions in ground and excited states. The interactions result in the decrease of fluorescence quantum yield. On the other hand, due to the spatial proximity of the chromophores, there is an energy transfer from the donor to the acceptor, resulting in emission only in the green region. Thus, dyad **11** has two fluorescence excitation peaks, from anthracene and BODIPY, but the emission occurs predominantly from BODIPY.

### 2.3. Singlet Oxygen Detection

**Figure 5:**
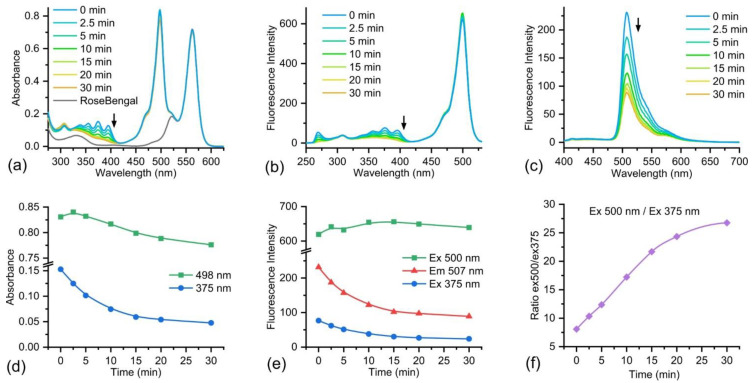
Absorption (**a**), fluorescence excitation (**b**), and fluorescence emission (**c**) spectra of BODIPY-DPA **11** in the course of singlet oxygen generation by RoseBengal. Spectra before and after green light irradiation for 2.5, 5, 10, 15, 20 and 30 min. Dynamics of absorption (**d**) and fluorescence (**e**) change at the specified wavelengths, as well as the ratio of fluorescence intensities when excited at 500 nm to 375 nm (**f**).

**Scheme 2:**
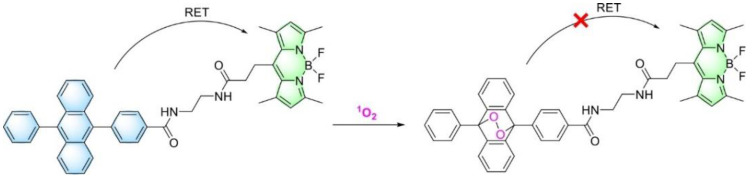
Reaction of BODIPY-DPA **11** with singlet oxygen.

To study the sensitivity of the BODIPY-DPA **11** dyad to singlet oxygen, we used the RoseBengal singlet oxygen generator, which has a maximum absorption at 562 nm and a shoulder at 520 nm (Figure 5a), allowing us to spectrally separate it from BODIPY and DPA. To the RoseBengal solution, BODIPY-DPA **11** was added, the mixture was irradiated with green light, and ^1^O_2_ generation was monitored by changes in the absorption and fluorescence spectra (Figure 5). One can see a decrease of characteristic anthracene absorption peaks in the 320–400 nm region during the reaction (Figure 5a). At the same time, the changes in the absorption region of BODIPY are not so significant. The similar changes are observed in the fluorescence excitation spectra (Figure 5b). In the fluorescence emission spectra when excited at 375 nm (Figure 5c), DPA fluorescence is virtually not observed. In the region of the BODIPY emission, the expected decrease of fluorescence intensity during the reaction is observed. The fluorescence remains considerable even after the completion of the process, since BODIPY also has an excitation maximum at 375 nm. A slight increase of both absorption and excitation peaks of BODIPY is clearly visible at the beginning of irradiation (Figure 5d,e). This can be attributed to the disruption of the interactions of BODIPY with DPA after the reaction with ^1^O_2_ (Scheme 2). The further decrease in BODIPY absorption could be explained either by photodegradation under the action of green light or by degradation caused by the generated singlet oxygen. In any case, the ratio of fluorescence intensities when excited at 500 nm to 375 nm increases significantly over time (3.3-fold, Figure 5f). Thus, this ratio can be used to monitor the dynamics of singlet oxygen generation.

## 3. Materials and Methods

The experimental details are presented in the Supplementary Materials.

## 4. Conclusions

In summary, in the present work, a BODIPY-DPA dyad was synthesized. In this dyad, the DPA unit acts as a highly specific oxygen sensor, whereas BODIPY acts as a reference fluorophore. The interchromophoric interactions between DPA and BODIPY lead, on the one hand, to a slight decrease in the fluorescence quantum yield and to the appearance of components with short fluorescence lifetime. On the other hand, due to the energy transfer from DPA to BODIPY, no DPA emission is observed in the fluorescence spectra; excitation of both DPA and BODIPY results in the fluorescence of the latter. When reacting with singlet oxygen, the intensity of the fluorescence excitation maximum associated with DPA decreases, while the excitation maximum of the BODIPY remains practically unchanged. This allows the BODIPY-DPA dyad to be used as a ratiometric sensor of singlet oxygen.

## Figures and Tables

**Scheme 1:**
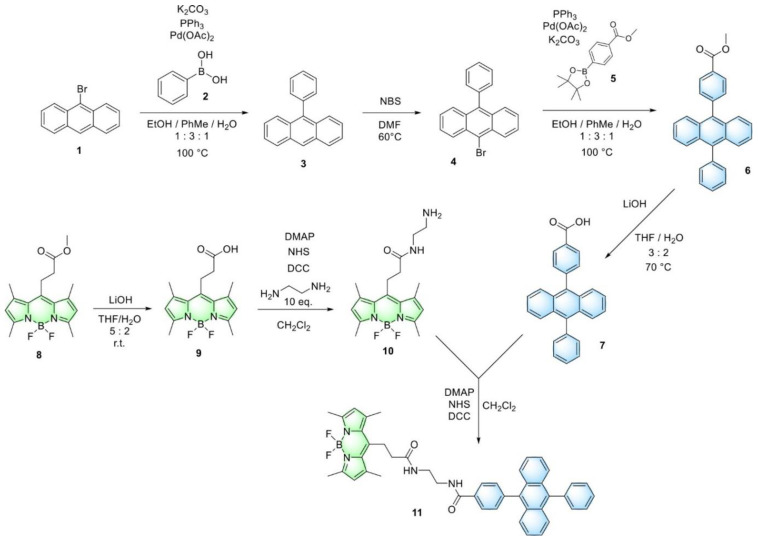
Synthesis of BODIPY-DPA dyad **11**.

**Table 1:** Photophysical properties of the synthesized compounds.

Compound	Solvent	λ_abs_ (nm)	ε (M^−1^ cm^−1^)	λ_em_ (nm)	*Φ*_f_	*Φ_ET_* ^6^	*Φ_ET_* ^7^
DPA-COOMe **6**	Dichloromethane	375	12,000	425	0.83 ^1^		
	Toluene	375	12,000	421	0.74 ^1^		
BODIPY-COOMe **8**	Dichloromethane	502	78,300	511	0.86 ^2^		
	Toluene	505	79,100	514	0.95 ^2^		
DPA-BODIPY **11**	Dichloromethane	375 (D) 502 (B)	-	424 (D) 512 (B)	0.76 (B) ^3^ 0.01 (D) ^4^ 0.60 (T) ^5^	0.99	0.72
	Toluene	375 (D) 504 (B)	-	425 (D) 515 (B)	0.90 (B) ^3^ 0.01 (D) ^4^ 0.66 (T) ^5^	0.99	0.65

^1^ Aerated solution of 9,10-diphenylanthracene in cyclohexane was used as a standard (*Φ*_f_ = 0.7)—$\Phi_{375}^{D}$. ^2^ Solution of fluorescein in 0.1N NaOH was used as a standard (*Φ*_f_ = 0.91)^. 3^ Emission of BODIPY part (excitation at 475 nm)—$\Phi_{475}^{BODIPY}$. ^4^ Emission of DPA part (excitation at 375 nm)—$\Phi_{375}^{DA}$. ^5^ Total emission (excitation at 375 nm)—$\Phi_{375}^{DA}+\Phi_{375}^{BODIPY}$. ^6^ The energy transfer efficiency was calculated using equation: *Φ_ET_* = 1 − $\Phi_{375}^{\mathrm{DA}}/\Phi_{375}^{D}$. ^7^ The energy transfer efficiency was calculated from dyads quantum yields measured with excitation at 375 nm and 475 nm using equation: $\Phi_{ET}=\frac{\Phi_{375}^{BODIPY}\left(\varepsilon_{375}^{DPA}+\varepsilon_{375}^{BODIPY}\right)}{\Phi_{475}^{BODIPY}\varepsilon_{375}^{DPA}}-\frac{\varepsilon_{375}^{BODIPY}}{\varepsilon_{375}^{DPA}}$. Details on this equation can be found in the Supplementary Materials.

**Table 2:** The results of analysis of fluorescence decay kinetics of compounds **6**, **8** and **11**.

Compound	Solvent	τ (ns) at 431 nm, λ_ex_ = 375 nm	τ (ns) at 511 nm, λ_ex_ = 375 nm	τ (ns) at 511 nm, λ_ex_ = 440 nm	Global Fitting	
					τ (ns), λ_ex_ = 375 nm	τ (ns), λ_ex_ = 440 nm
DPA-COOMe **6**	Dichloromethane	5.25				
	Toluene	4.57				
BODIPY-COOMe **8**	Dichloromethane		5.3	5.34		
	Toluene		4.9	4.94		
DPA-BODIPY **11**	Dichloromethane	5.24 (37%) 1.54 (8%) 0.07 (55%)	5.28 (79%) 0.96 (21%)	5.3 (74%) 0.96 (26%)	5.29 0.98 0.05	5.26 0.63
	Toluene	4.8 (3%) 1.74 (7%) 0.06 (90%)	4.96 (84%) 0.80 (16%)	4.93 (74%) 0.74 (26%)	4.92 1.27 0.06	4.93 0.5

## Data Availability

Not applicable.

## Supplementary Materials

The following supporting information can be downloaded at: https://www.mdpi.com/article/10.3390/molecules27249060/s1.
