# Supplementary material for: Ratiometric Singlet Oxygen Sensor Based on BODIPY-DPA Dyad
Source: Molecules. 2022 Dec 19;27(24):9060. doi: 10.3390/molecules27249060 (PMC9780792; doi:10.3390/molecules27249060)
Supplement: Supplementary file 1 [file molecules-27-09060-s001.zip › molecules-2111857-supplementary.pdf]

Supplementary Materials

Ratiometric singlet oxygen sensor based on BODIPY-DPA dyad

Alexey A. Pakhomov[1,2,*], Anastasia S. Belova[2], Arevik G. Khchoyan[2,3], Yuriy N. Kononevich[2], Dmitriy S. Ionov[4], Anastasiya Yu. Frolova[1,2], Mikhail V. Alfimov[4,5], Vladimir I. Martynov[1] and Aziz M. Muzafarov[2,6]

[1] M.M. Shemyakin and Yu.A. Ovchinnikov Institute of Bioorganic Chemistry, Russian Academy of Sciences, 117997 Moscow, Russian Federation

[2] A.N. Nesmeyanov Institute of Organoelement Compounds, Russian Academy of Sciences, 119991 Moscow, Russian Federation

[3] Mendeleev University of Chemical Technology of Russia, 125047 Moscow, Russia

[4] Photochemistry Center, FSRC “Crystallography and Photonics”, Russian Academy of Sciences, 119421 Moscow, Russian Federation

[5] Moscow Institute of Physics and Technology (State University), 141707 Dolgoprudny, Russian Federation

[6] N.S. Enikolopov Institute of Synthetic Polymeric Materials, Russian Academy of Sciences, 117393 Moscow, Russian Federation

* Correspondence: alpah@mail.ru

Table of Contents

1. General Methods and Materials
2. Synthesis
3. Spectral Data
4. Absorption and Emission Spectra
5. References

1. General Methods and Materials

All solvents were purified before use. Dichloromethane was distilled over phosphorus pentoxide. Toluene, ethanol, chloroform, methanol, acetone and ethylacetate were distilled. 9-Bromoanthracene, $Pd(OAc)_2$, triphenylphosphine, phenylboronic acid, N-bromosuccinimide (NBS), ethylenediamine, N,N′-dicyclohexylcarbodiimide (DCC), N-hydroxysuccinimide (NHS) and 4-dimethylaminopyridine (DMAP) were purchased from Acros Organics. The reactions were monitored by thin-layer chromatography (TLC) using Fluka silica gel (60 F 254) plates (0.25 mm). Column chromatography was carried out using Merck 60 (230–400 mesh) silica gel. Visualization was made with UV light. IR spectra were obtained using an IR spectrometer with a Fourier transformer Shimadzu IRTracer-100 (Japan). The samples were prepared by pressing KBr pellets. ^{1}H, ^{13}C and ^{19}F NMR spectra were recorded on a Bruker Avance 400 (400 MHz; Germany) spectrometer. Chemical shifts are reported relative to chloroform (δ=7.25 ppm) for ^{1}H NMR and chloroform (δ=77.00 ppm) for ^{13}C NMR. High-resolution mass spectra (HRMS) were measured using a Bruker micrOTOF II instrument with electrospray ionization (ESI) (Germany).

The **absorption spectra** were recorded on a Shimadzu UV-1900 spectrophotometer (Japan). The **fluorescence spectra** were acquired using Shimadzu RF-6000 spectrofluorophotometer (Japan). Spectroscopic grade solvents (Aldrich) were used in UV-vis absorption and fluorescence measurements. **Fluorescence decay curves** were obtained using spectrofluorometer Fluotime 300 (Picoquant). LDH-D-C-375 (λ_{ex}=375 nm) and LDH-P-C-440 (λ_{ex}=440 nm) were used as excitation sources. Data fitting was performed using Easytau2 (Picoquant) software using the following model for multiexponential fitting of experimental data:

$$\mathrm{Dec}(t)=\left[\int_{-\infty}^{t} dt'\left[\mathrm{IRF}(t-Shift_{IRF})-Bkgr_{IRF}\right]\left[\sum_{i=1}^{n_{Exp}} A_i\, e^{-\frac{t-t'}{\tau_i}}+A_{Scatt}\,\delta(t-t')\right]\right]+Bkgr_{Dec}$$

$$I_m = A_m\tau_m \qquad \tau_{Av\,Int} = \sum_{\substack{i=1\\ I_i>0}}^{n_{Exp}} I_i\tau_i \Big/ \sum_{\substack{i=1\\ I_i>0}}^{n_{Exp}} I_i$$

FWHM of instrument response function (IRF) is about 180 ps and is determined manly by detection system (laser pulse FWHM is about 40 ps). According to literature [1] estimation for exponential lifetimes in deconvolution procedure used is about 0.1 FWHW IRF, the same estimation is proposed by equipment manufacturer. Thus, in our case lifetime accuracy is about 20 ps and this is upper estimation. More precise experimental estimation may be performed using the following method [2]. Two pump pulse profiles are measured, a length of time corresponding to the time needed to collect an ordinary decay curve being allowed to elapse between the two measurements. Deconvolution of one pump pulse profile with the other will

yield a "decay" time, which is a measure of the time resolution of the instrument. In our case error obtained using this method is about 6 ps with about 10 minutes between measurements.

To choose appropriative number of exponential terms we have used criteria that χ^2 is in range 0.8 - 1.3 [3] and uniformity of residuals distribution and auto-correlation function around zero.

We have used two methods for estimation of **excitation energy transfer efficiency** Φ_{ET}. The detailed discussion on this topic can be found in literature [4]. In method 1 efficiency is estimated using fluorescence quantum yields:

$$\Phi_{\mathrm{ET}} = \mathrm{k}_{ET}\tau_{DA} = 1 - \frac{\tau_{DA}}{\tau_D} = 1 - \frac{k_D^f \tau_{DA}}{k_D^f \tau_D} = 1 - \frac{\Phi_{375}^{\mathrm{DA}}}{\Phi_{375}^{\mathrm{D}}}$$

Where τ_{DA} – fluorescence lifetime of donor in dyad, τ_{D} – fluorescence lifetime of free donor, k_{ET} - rate constant of energy transfer, Φ_{375}^{DA} – fluorescence quantum yield of donor in dyad (quantum yield of Emission of DPA part (D) obtained with excitation at 375 nm in table 1), Φ_{375}^{D} – fluorescence quantum yield of free donor (quantum yield of Emission of DPA-COOMe in table 1), k_D^f – radiation rate constant of donor.

It is worth to note that this method relays on fact that quenching of donor in excited state is only due to the energy transfer. All excitation energy lost by donor will be received by acceptor. In general, it is not always valid assumption. To more precisely estimate $\boldsymbol{\Phi_{ET}}$ lets consider the following equation which describes integral fluorescence intensity of acceptor unit when the system is excited at donor wavelength 375nm. In this case fluorescence arises due to direct acceptor excitation and excitation through donor:

$$\mathrm{I}_{375} = \Phi_{375}^{BODIPY}(D_{375}^{BODIPY} + D_{375}^{DPA})I = \Phi_{475}^{BODIPY}(D_{375}^{BODIPY} + \Phi_{\mathrm{ET}} D_{375}^{DPA})I$$

Where $\boldsymbol{I}$ – excitation light intensity, $\boldsymbol{\Phi_{375}^{BODIPY}}$ – BODIPY unit quantum yield upon excitation at 375 nm (quantum yield of Emission of BODIPY part obtained with excitation at 375 nm, difference between total quantum yield (T) and Emission of DPA part (D) in table 1), $\boldsymbol{\Phi_{375}^{BODIPY}}$ – BODIPY unit quantum yield upon excitation at 475 nm (quantum yield of Emission of BODIPY part obtained with excitation at 475 nm (B) in table 1), $\mathbf{D_{375}^{BODIPY}}$ – optical density at 375 nm related to BODIPY unit, $\mathbf{D_{375}^{DPA}}$ – optical density at 375 nm related to DPA unit. From this equation the following equation for $\boldsymbol{\Phi_{ET}}$ can be obtained.

$$\Phi_{\mathrm{ET}} = \frac{\Phi_{375}^{BODIPY}(D_{375}^{DPA} + D_{375}^{BODIPY})}{\Phi_{475}^{BODIPY} D_{375}^{DPA}} - \frac{D_{375}^{BODIPY}}{D_{375}^{DPA}} = \frac{\Phi_{375}^{BODIPY}(\varepsilon_{375}^{DOA} + \varepsilon_{375}^{BODIPY})}{\Phi_{475}^{BODIPY}\varepsilon_{375}^{DPA}} - \frac{\varepsilon_{375}^{BODIPY}}{\varepsilon_{375}^{DPA}}$$

where $\boldsymbol{\varepsilon_{375}^{BODIPY}}$ – extinction at 375 nm related to BODIPY unit, $\boldsymbol{\varepsilon_{375}^{DPA}}$ – extinction at 375 nm related to DPA unit.

For the **singlet oxygen generation** experiments, DPA-BODIPY was added to RoseBengal solution in DMF in microtubes. Samples were irradiated using a diode array with an emission maximum at 520 nm (FWHM 60 nm). The irradiation intensity was adjusted so that the process of the sensor transformation was completed in approximately 30 minutes. Samples of 100 µl were taken at specified time intervals and absorption spectra were measured using Varian Cary 50 Bio spectrophotometer (Australia), then samples were diluted to 1 mL and fluorescence spectra were measured using Varian Cary Eclipse spectrofluorophotometer (Australia).

2. Synthesis

*9-Ph-Anth (**3**).* A mixture of 9-bromoanthracene **1** (2.17 g, 8.4 mmol), K_2CO_3 (2.32 g, 16.8 mmol), $Pd(OAc)_2$ (0.038 g, 2 mol%), PPh_3 (0.089 g, 4 mol%) and phenylboronic acid **2** (1.54 g, 12.6 mmol) was placed in a two-necked round-bottom flask under argon atmosphere and then deaerated mixture of ethanol/toluene/water (1:3:1, 50 mL) was added. The reaction mixture was reflux for 24 h. After that the mixture was cooled down to room temperature, poured into water (100 mL) and then extracted with dichloromethane (3 times by 50 mL). The combined organic layer was washed with brine, separated by diluted funnel and dried with anhydrous Na_2SO_4. The solvent was removed by rotor evaporator and the product was purified by column chromatography on silica using a mixture of hexane:dichloromethane as an eluent. The product **3** was obtained as a light-yellow powder. **Yield**: 90%. **^{1}H NMR** (400 MHz, $CDCl_3$): δ 7.36-7.01 (m, 2H), 7.46-7.51 (m, 4H), 7.56-7.63 (m, 3H), 8.07 (d, J = 8.5 Hz, 2H), 8.52 (s, 1H). **^{13}C NMR** (101 MHz, $CDCl_3$): δ 125.0, 125.3, 126.5, 126.8, 127.4, 128.3, 128.3, 130.2, 131.2, 131.3, 136.9, 138.7. **IR** (KBr, cm^{-1}): 3056, 3033, 1627, 1444, 1413, 1385, 1358, 1347, 1166, 1074, 1018, 957, 934, 880, 847, 791, 756, 738, 703, 639, 612, 552.

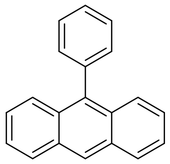

*9-Ph-10-Br-Anth (**4**).* A solution of 9-phenylanthracene **3** (1 g, 3.9 mmol) and N-bromosuccinimide (0.7 g, 3.9 mmol) in dry chloroform (20 mL) was stirred at 60 °C for 3 h under argon atmosphere. After that the mixture was cooled down to room temperature, solvent was removed under reduced pressure and the product was re-dissolved in acetone and added to methanol. The precipitate was filtered off and washed with methanol to give 9-bromo-10-phenylanthracene **4**. **Yield**: 95%. **^{1}H NMR** (400 MHz, $CDCl_3$): δ 7.38-7.42 (m, 4H), 7.56-7.67 (m, 5H), 7.66 (d, J = 9.1 Hz, 2H), 8.62 (d, J = 9.6 Hz, 2H). **^{13}C NMR** (101 MHz, $CDCl_3$): δ 122.7, 125.5, 126.9, 127.4, 127.7, 127.8, 128.4, 130.2, 131.0, 131.1, 137.8, 138.4. **IR** (KBr, cm^{-1}): 3077, 3056, 1441, 1344, 1262, 1153, 1071, 1031, 938, 881, 759, 704, 643, 614, 579.

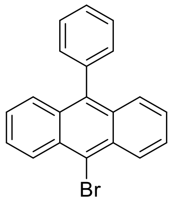

*$Me_4C_2O_2$BPhCOOMe (**5**).* It was synthesized by the method described earlier *[5]*.

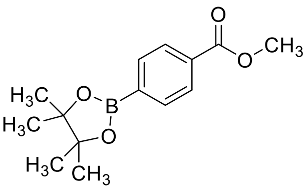

*DPA-COOMe (**6**).* A mixture of 9-bromo-10-phenylanthracene **4** (0.17 g, 0.5 mmol), K_2CO_3 (2.32 g, 1 mmol), $Pd(OAc)_2$ (0.0022 g, 2 mol%), PPh_3 (0.0053 g, 4 mol%) and $Me_4C_2O_2$BPhCOOMe **5** (0.16 g, 0.6 mmol) was placed in a two-necked round-bottom flask under argon atmosphere and then deaerated mixture of ethanol/toluene/water (1:3:1, 5 mL) was added. The reaction mixture was reflux for 96 h. After that the mixture was cooled down to room temperature, poured into water (20 mL) and then extracted with dichloromethane (3 times by 20 mL). The combined organic layer was washed with brine, separated by diluted funnel and dried with anhydrous Na_2SO_4. The solvent was removed by rotor evaporator and the product was purified by column chromatography on silica using toluene as an eluent. The product **6** was obtained as a white powder. **Yield**: 84%. **^{1}H NMR** (400 MHz, $CDCl_3$): δ 4.02 (s, 3H, OCH_3), 7.32-7.36 (m, 4H, Ar), 7.47-7.49 (m, 2H, Ar), 7.56-7.63 (m, 7H, Ar), 7.69-7.73 (m, 2H, Ar), 8.29 (d, *J* = 8.1 Hz, 2H), **^{13}C NMR** (101 MHz, DMSO-d_6): δ 52.3, 125.1, 125.6, 126.5, 127.1, 127.6, 128.4, 129.4, 129.5, 129.7, 129.8, 131.2, 131.5, 135.7, 137.7, 138.8, 144.3, 167.1. **IR** (KBr, cm^{-1}): 3034, 2949, 1721, 1606, 1599, 1440, 1433, 1393, 1372, 1291, 1278, 1193, 1172, 1112, 1100, 1024, 1019, 1014, 964, 942, 913, 881, 851, 825, 772, 753, 696, 670, 611, 517, 495. **HRMS** (ESI) *m/z* calcd. for $C_{28}H_{21}O_2$ [$(M+H)^+$]: 389.1536, found 389.1523.

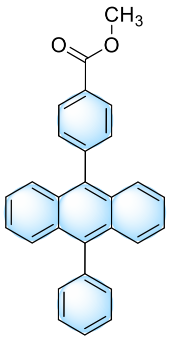

*DPA-COOH (**7**).* A mixture of DPA-COOMe **6** (0.14 g, 0.36 mmol), KOH (0.1 g, 1.8 mmol) and THF/H_2O (3:2, 5 mL) was stirred under reflux for 5 h. After that the mixture was cooled down to room temperature and acidified with 10% HCl. The precipitate was filtered off and washed with water. The product **7** was obtained as a white powder. **Yield**: 90%. **^{1}H NMR** (400 MHz, DMSO-d_6): δ 7.43-7.48 (m, 6H, Ar), 7.52-7.55 (m, 2H, Ar), 7.58-7.62 (m, 5H, Ar), 7.65-7.69 (m, 2H, Ar), 8.22 (d, *J* = 7.8 Hz, 2H), 13.17 (s, 1H, COOH). **IR** (KBr, cm^{-1}): 3424, 3062, 2959, 2925, 2855, 1642, 1593, 1547, 1438, 1404, 1391, 1261, 1175, 1097, 1052, 1027, 943, 826, 797, 789, 768, 753, 702, 665, 611, 473. **HRMS** (ESI) *m/z* calcd. for $C_{27}H_{18}O_2$ [(M)]: 374.1301, found 374.1291.

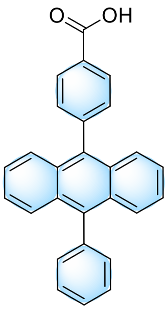

BODIPY-COOH (9). It was synthesized by the method described earlier by us *[6]*.

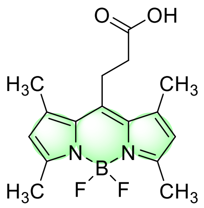

BODIPY-DPA (11). A mixture of BODIPY-COOH **9** (0.2 g, 0.625 mmol), ethylenediamine

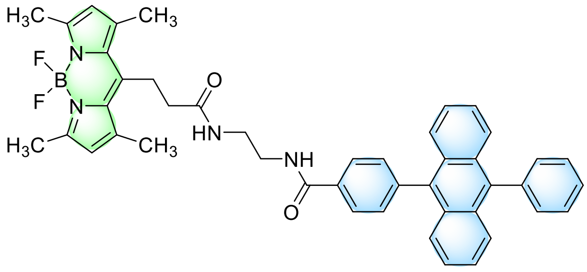

(0.375 g, 6.25 mmol), N,N′-dicyclohexylcarbodiimide (0.155 g, 0.75 mmol), N-hydroxysuccinimide (0.086 g, 0.75 mmol) and 4-dimethylaminopyridine (0.005 g) in dichloromethane (10 mL) was stirred at room temperature for 24 h. After the reaction was complete a solvent was removed by rotor evaporator. The residue was purified by column chromatography on silica with a mixture chloroform:methanol (1:1) as an eluent. Product **10** was obtained as an orange oil. Then, a mixture of BODIPY-NH_2 **10** (0.09 g, 0.25 mmol), DPA-COOH **7** (0.093 g, 0.25 mmol), N,N′-dicyclohexylcarbodiimide (0.062 g, 0.3 mmol), N-hydroxysuccinimide (0.035 g, 0.3 mmol) and 4-dimethylaminopyridine (0.002 g) in dichloromethane (5 mL) was stirred at room temperature for 24 h. After the reaction was complete a solvent was removed by rotor evaporator. The residue was purified by column chromatography on silica with a mixture ethylacetate:chloroform (3:1) as an eluent. Product **10** was obtained as an orange oil. **Yield**: 67 %. **^{1}H NMR** (400 MHz, $CDCl_3$): δ 2.43 (s, 6H, CH_3), 2.50 (s, 6H, CH_3), 2.54 (m, 2H, CH_2), 3.35 (m, 2H, CH_2), 3.60 (m, 4H, CH_2), 6.03 (s, 2H, BODIPY-CH), 6.60 (m, 1H, NH), 7.29-7.35 (m, 5H, Ar, NH), 7.47 (d, 2H, J = 7.2 Hz, Ar), 7.56-7.63 (m, 7H, Ar), 7.70 (d, 2H, J = 8.3 Hz, Ar), 8.05 (d, 2H, J = 7.8 Hz, Ar). **^{13}C NMR** (101 MHz, DMSO-d_6): δ 14.1, 16.1, 24.0, 36.5, 38.4, 38.9, 121.8, 125.6, 125.7, 126.2, 126.5, 127.7, 127.9, 128.7, 129.0, 129.2, 130.6, 130.9, 130.9, 134.0, 135.8, 137.0, 138.1, 141.1, 141.2, 145.7, 153.4, 166.3, 170.4. **^{19}F NMR** (376 MHz, DMSO-d_6) δ -143.82. **IR** (KBr, cm^{-1}): 3422, 2964, 2925, 2854, 1651, 1633, 1551, 1509, 1473, 1459, 1409, 1372, 1309, 1259, 1224, 1201, 1160, 1115, 1084, 1028, 985, 769, 757, 704, 667, 481. **HRMS** (ESI) *m/z* calcd. for $C_{45}H_{41}NaBF_2N_4O_2$ $[(M+Na)^+]$: 741.3190, found 741.3182.

3. Spectral Data

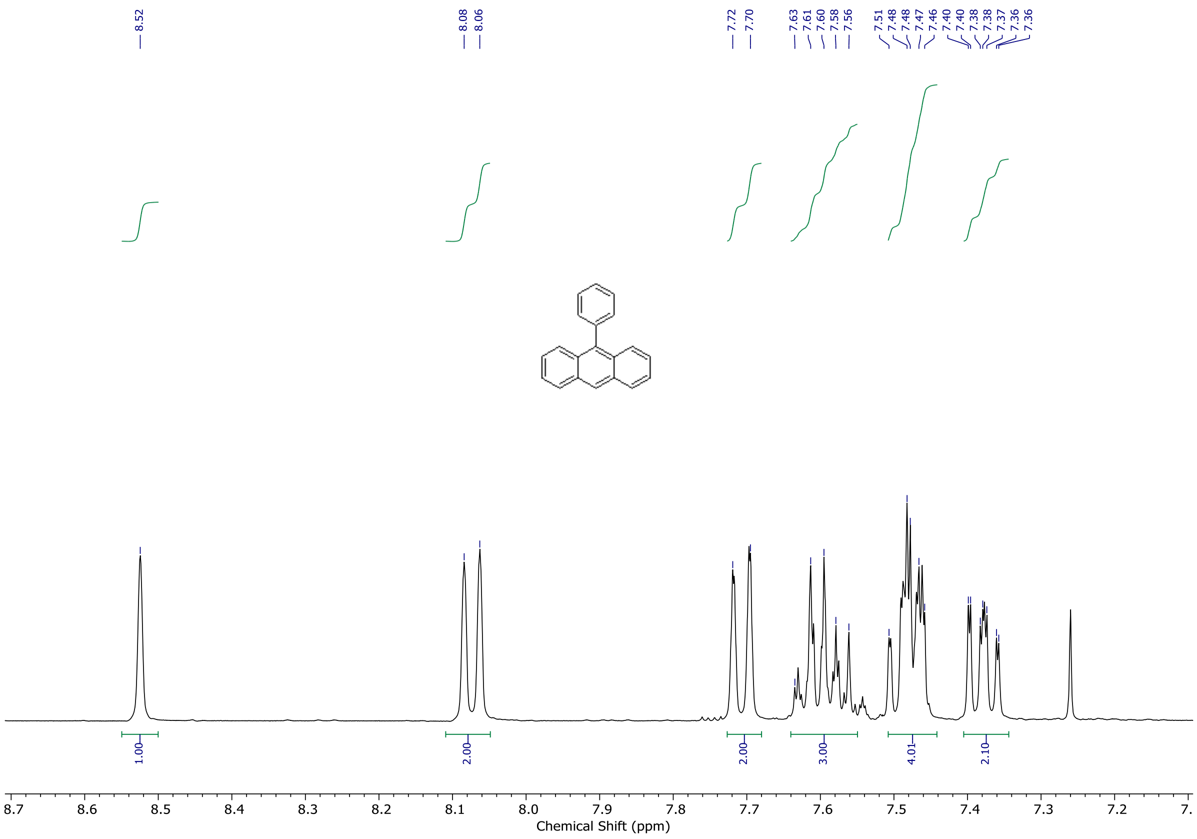

Figure S1. ^{1}H NMR spectrum of **3** in $CDCl_3$.

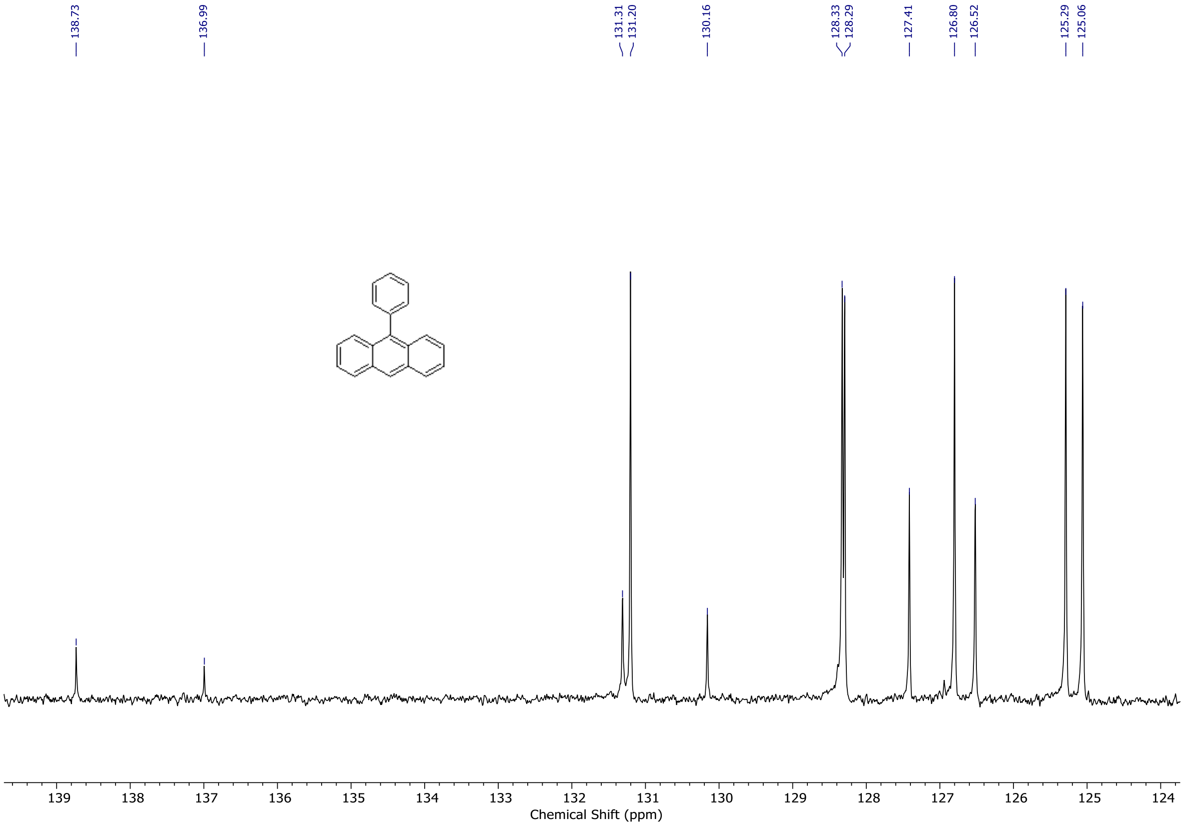

Figure S2. ^{13}C NMR spectrum of **3** in $CDCl_3$.

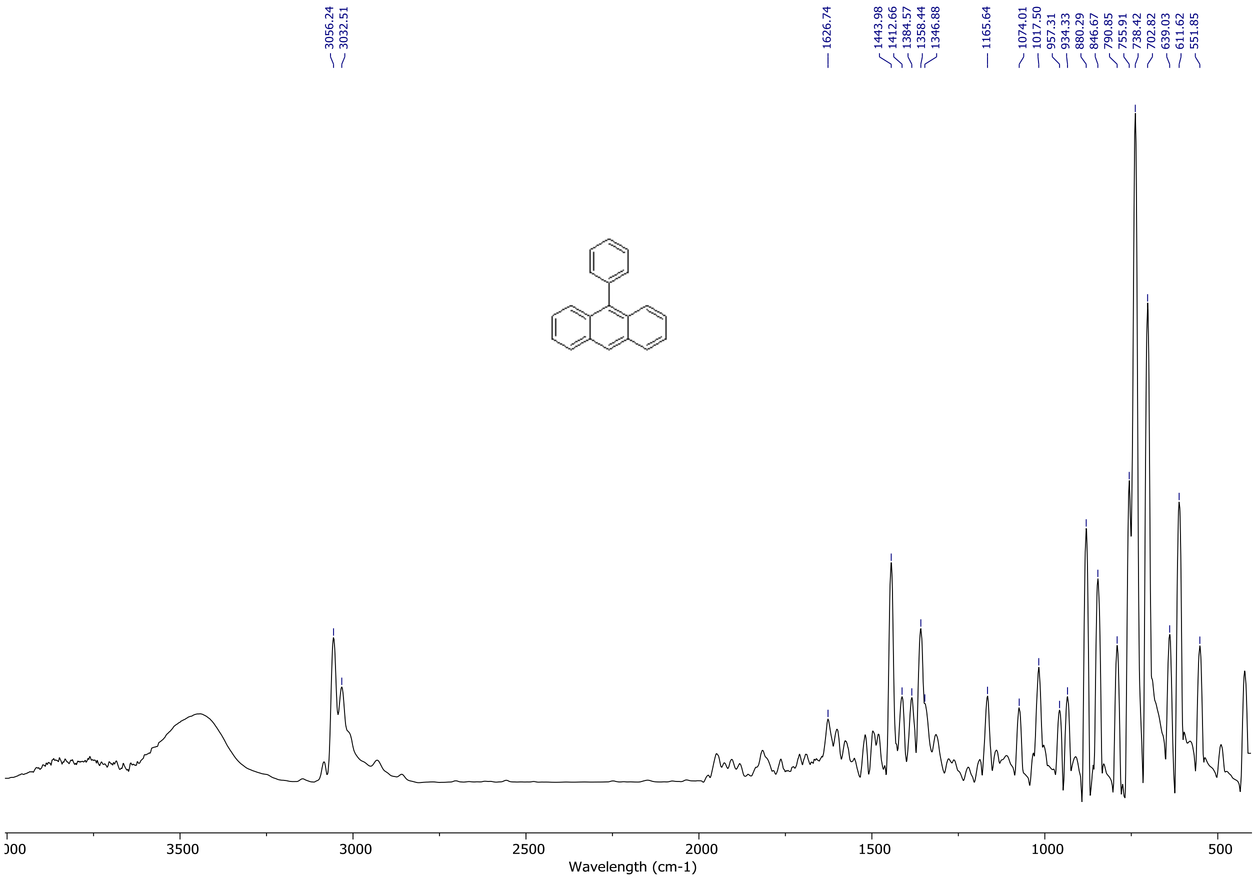

Figure S3. IR spectrum of **3**.

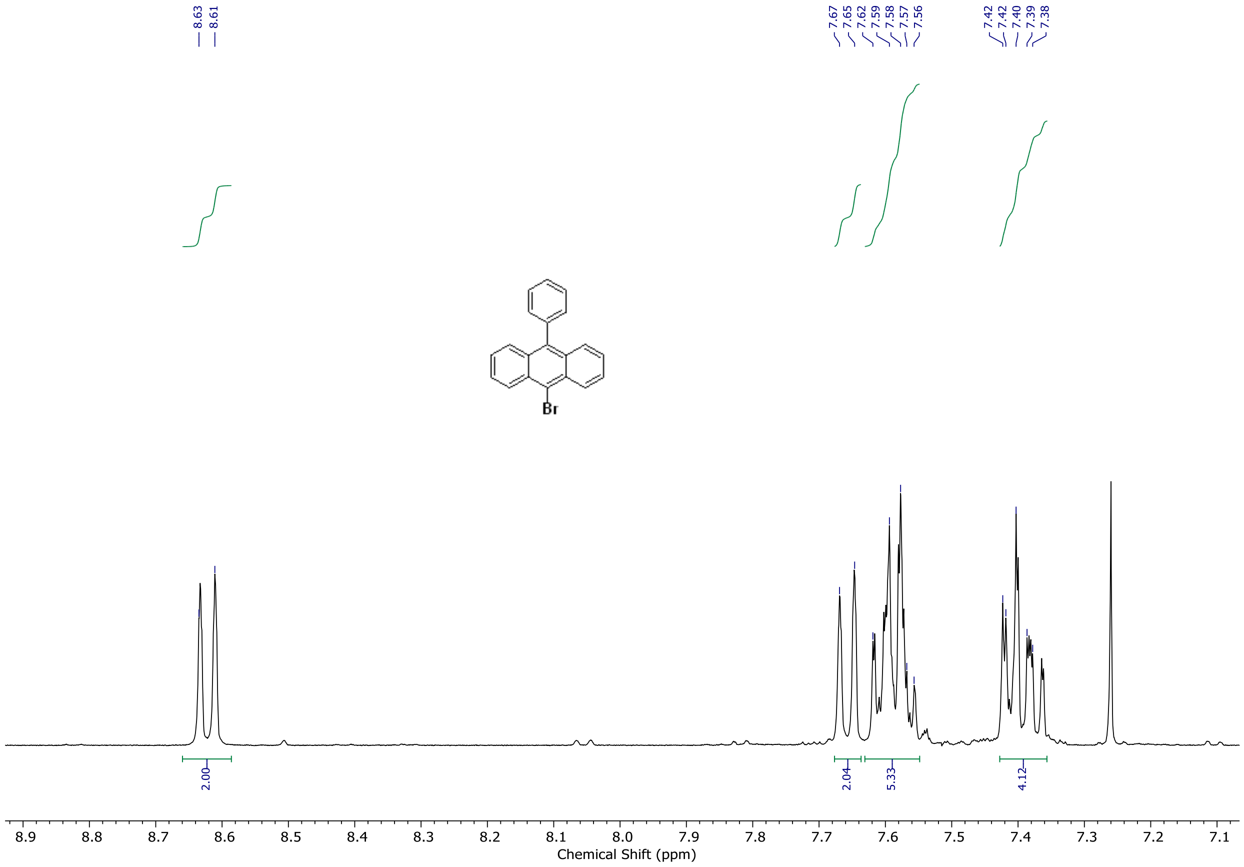

Figure S4. ^{1}H NMR spectrum of **4** in $CDCl_3$.

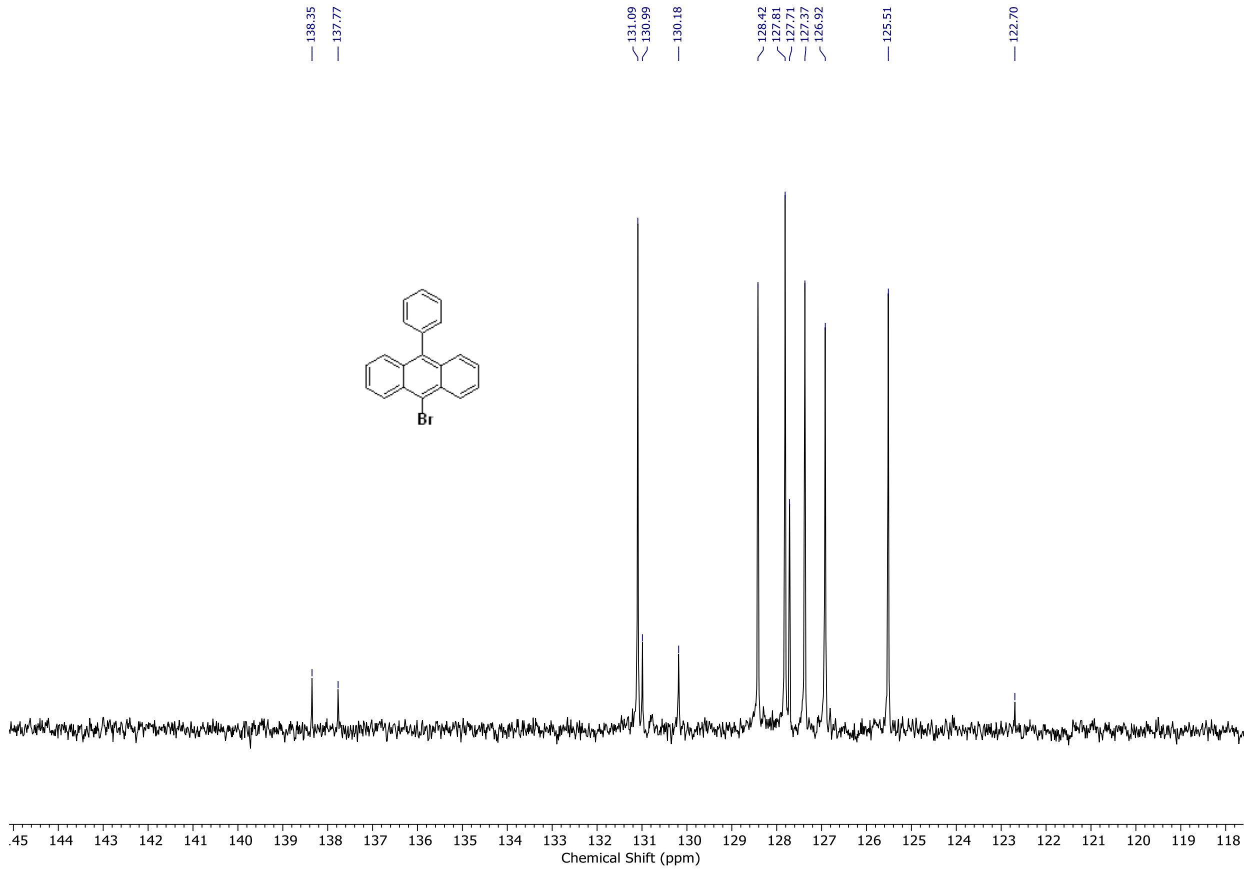

Figure S5. ^{13}C NMR spectrum of **4** in $CDCl_3$.

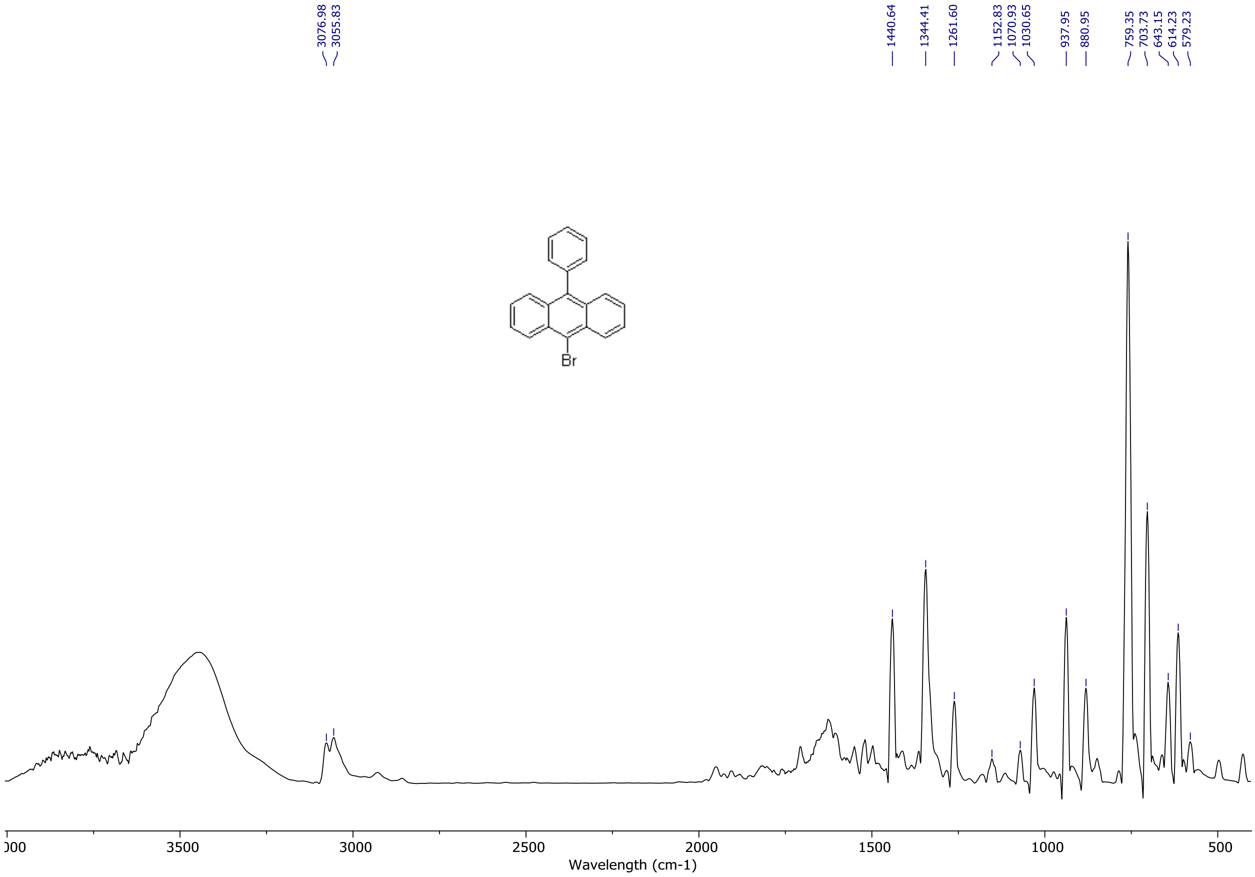

Figure S6. IR spectrum of **4**.

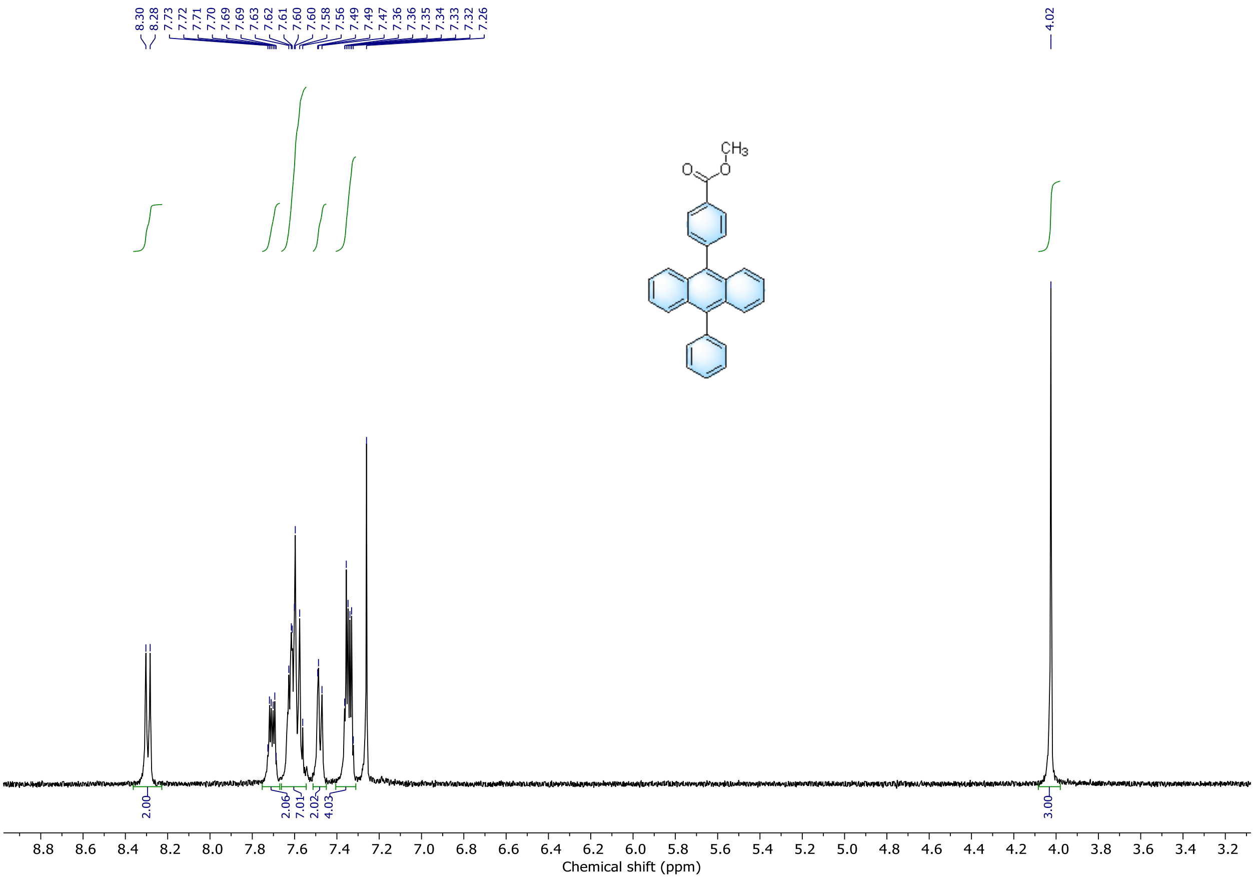

Figure S7. ^{1}H NMR spectrum of **6** in $CDCl_3$.

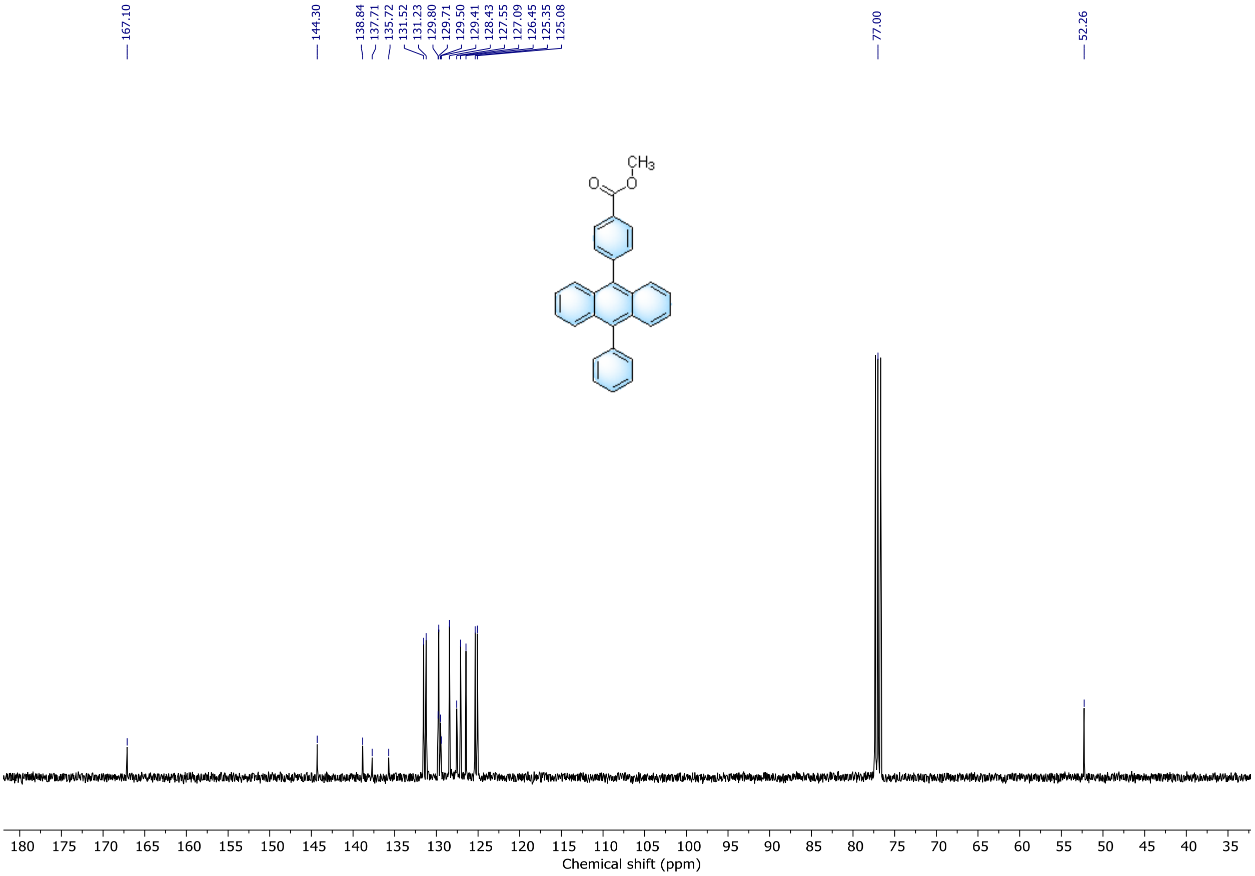

Figure S8. ^{13}C NMR spectrum of **6** in $CDCl_3$.

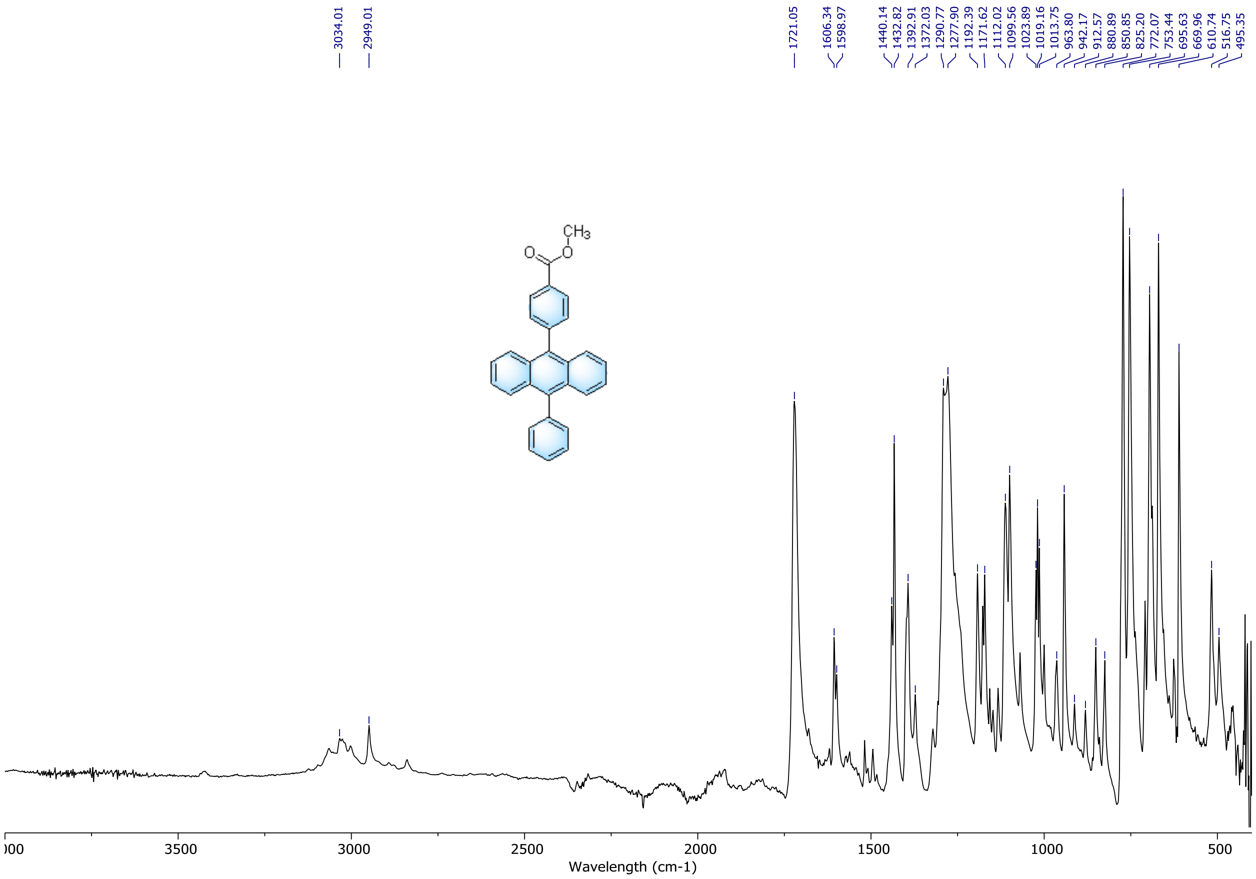

Figure S9. IR spectrum of **6**.

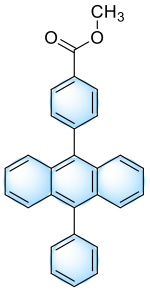

m/z calcd. for $C_{28}H_{21}O_2$ [$(M+H)^+$]: 389.1536

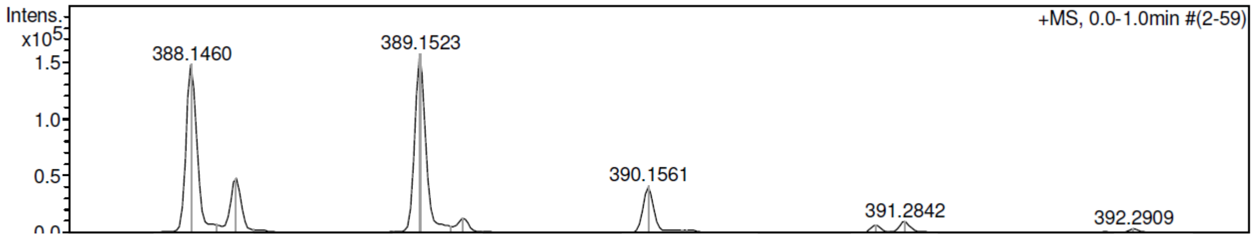

Figure S10. Mass-spectrum of **6**.

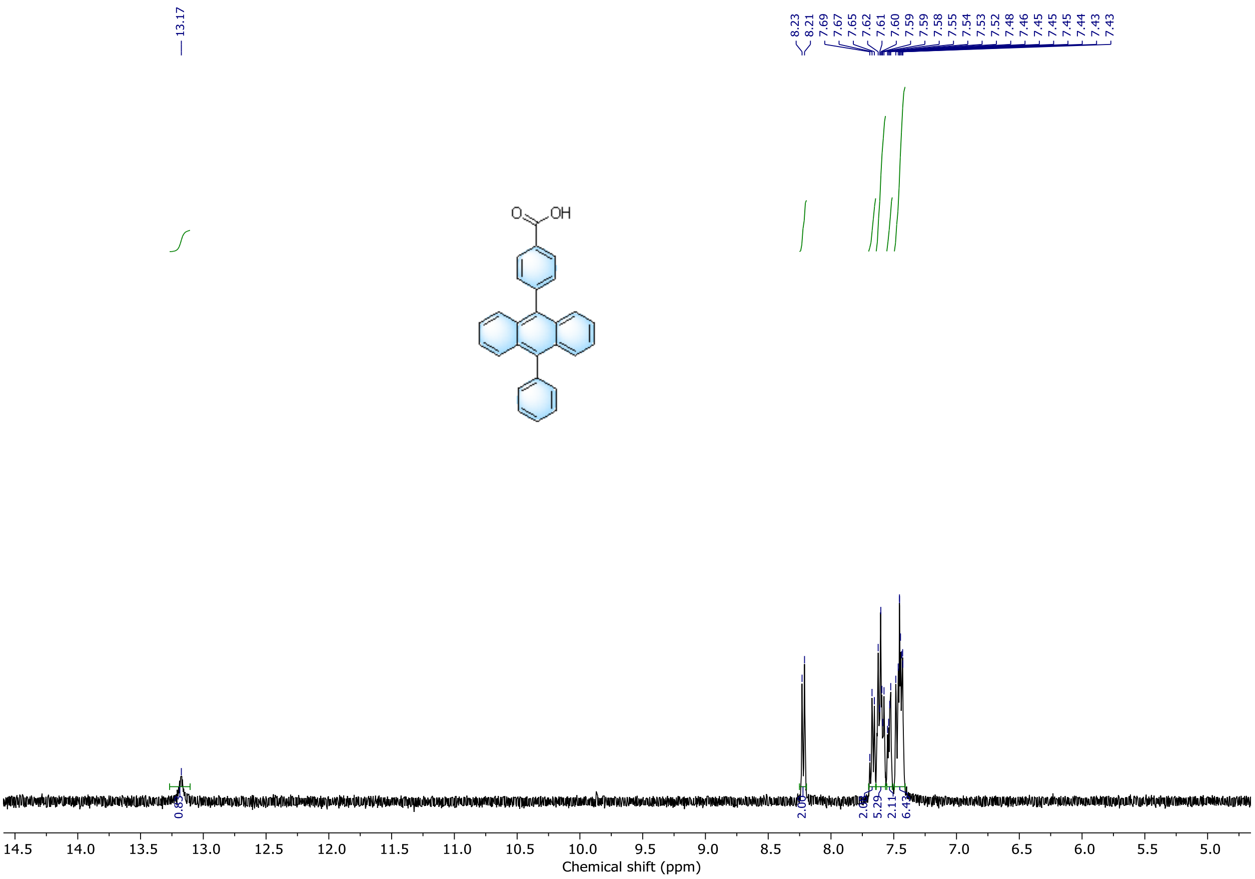

Figure S11. ^{1}H NMR spectrum of **7** in DMSO-d_6.

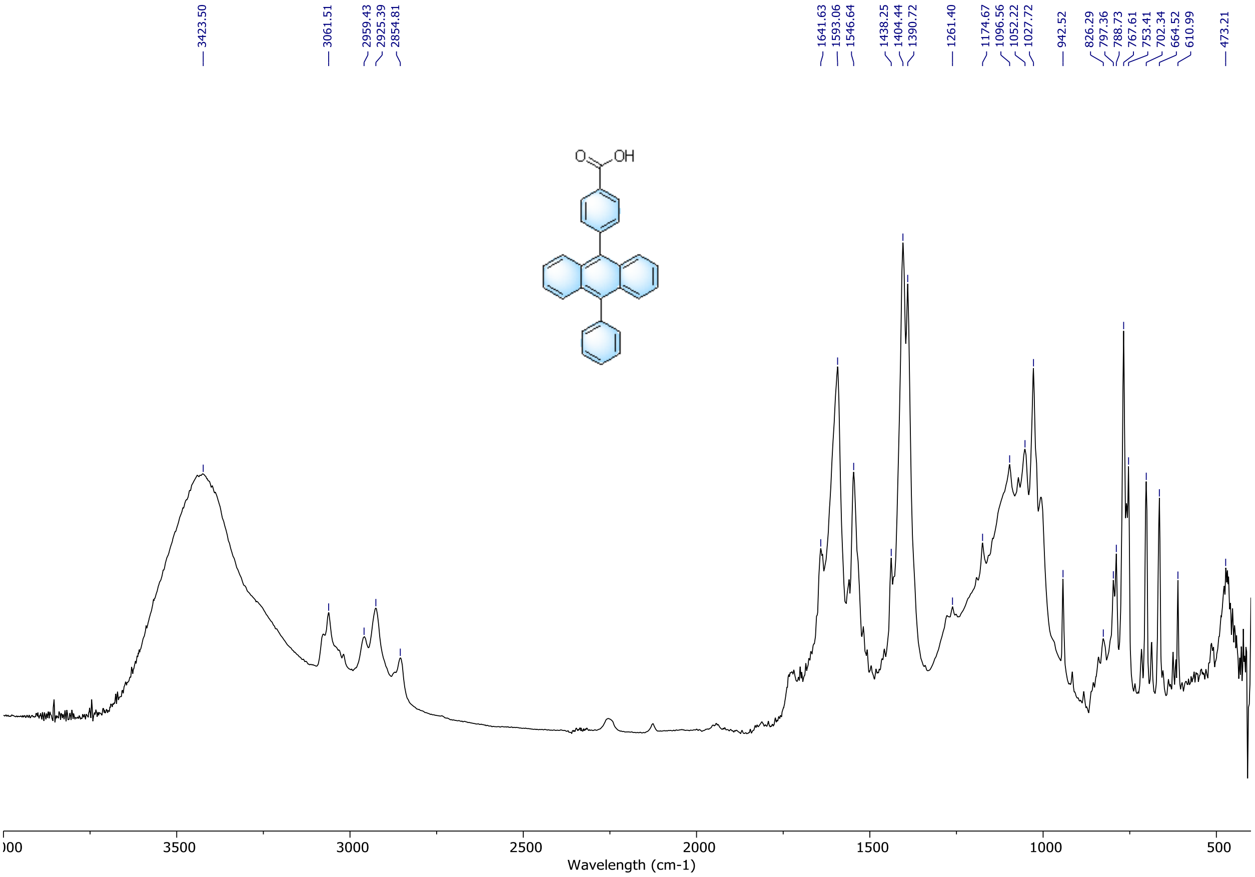

Figure S12. IR spectrum of **7**.

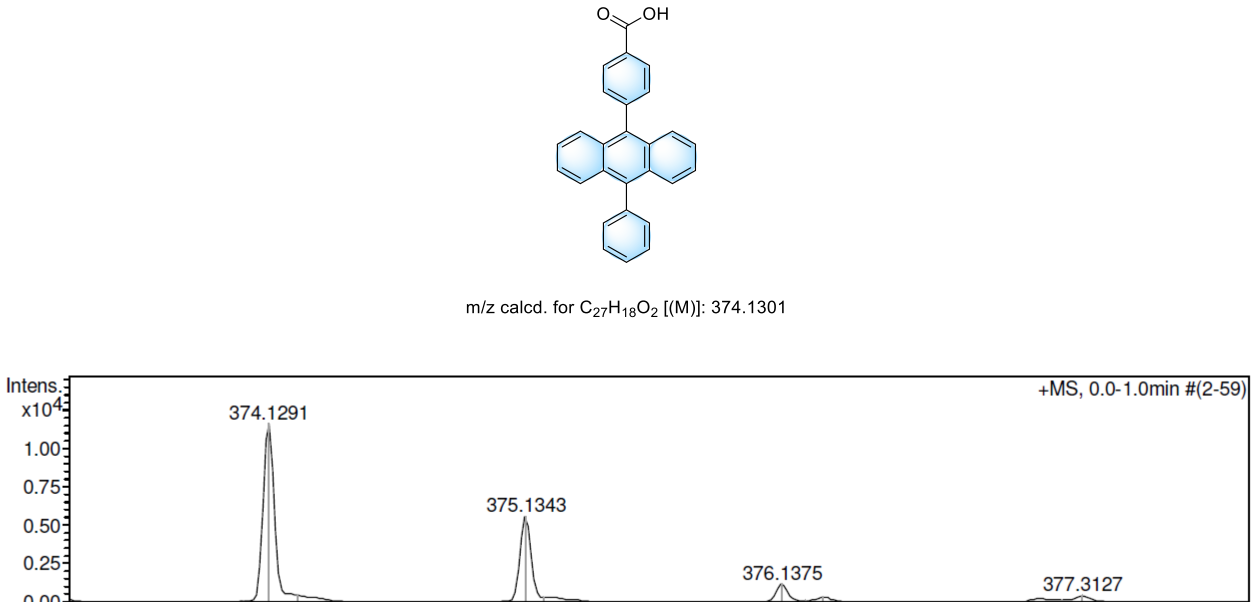

Figure S13. Mass-spectrum of **7**.

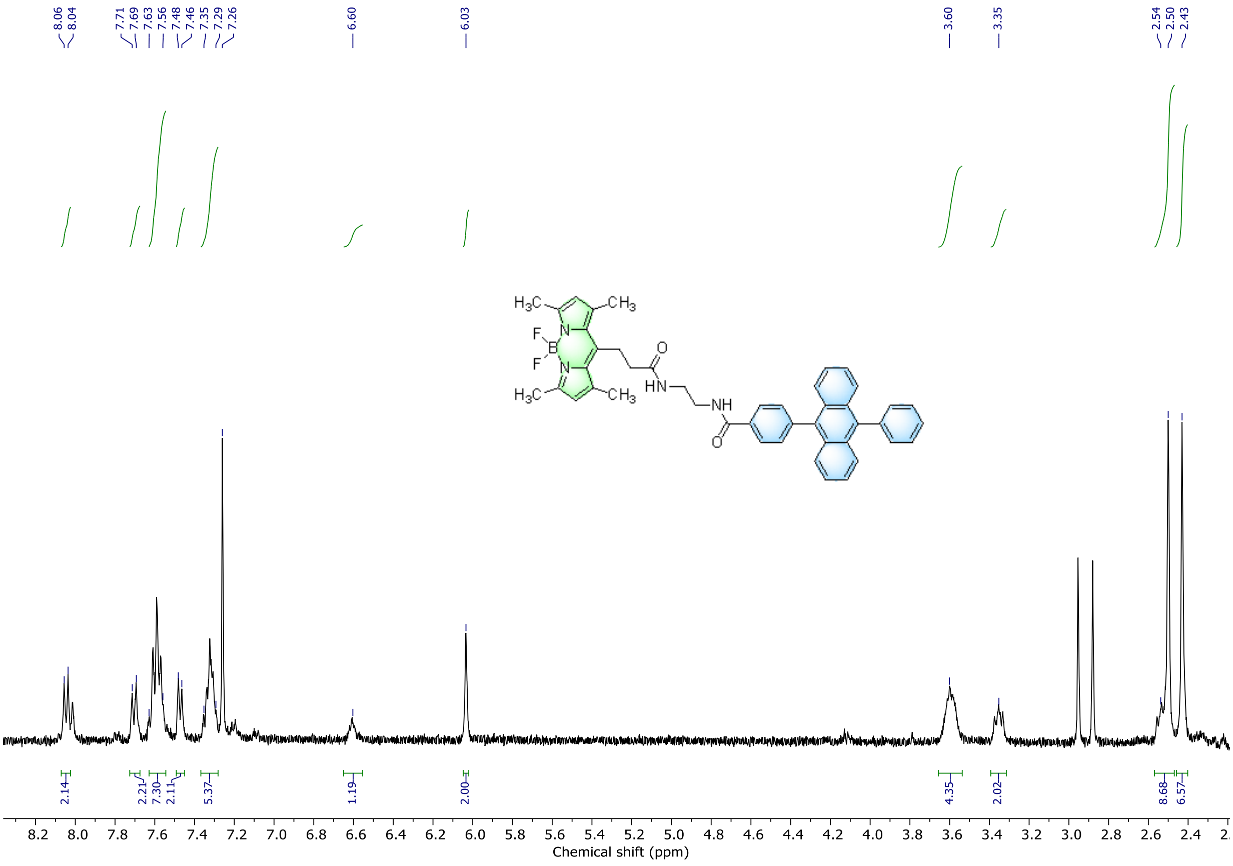

Figure S14. ^{1}H NMR spectrum of **11** in $CDCl_3$.

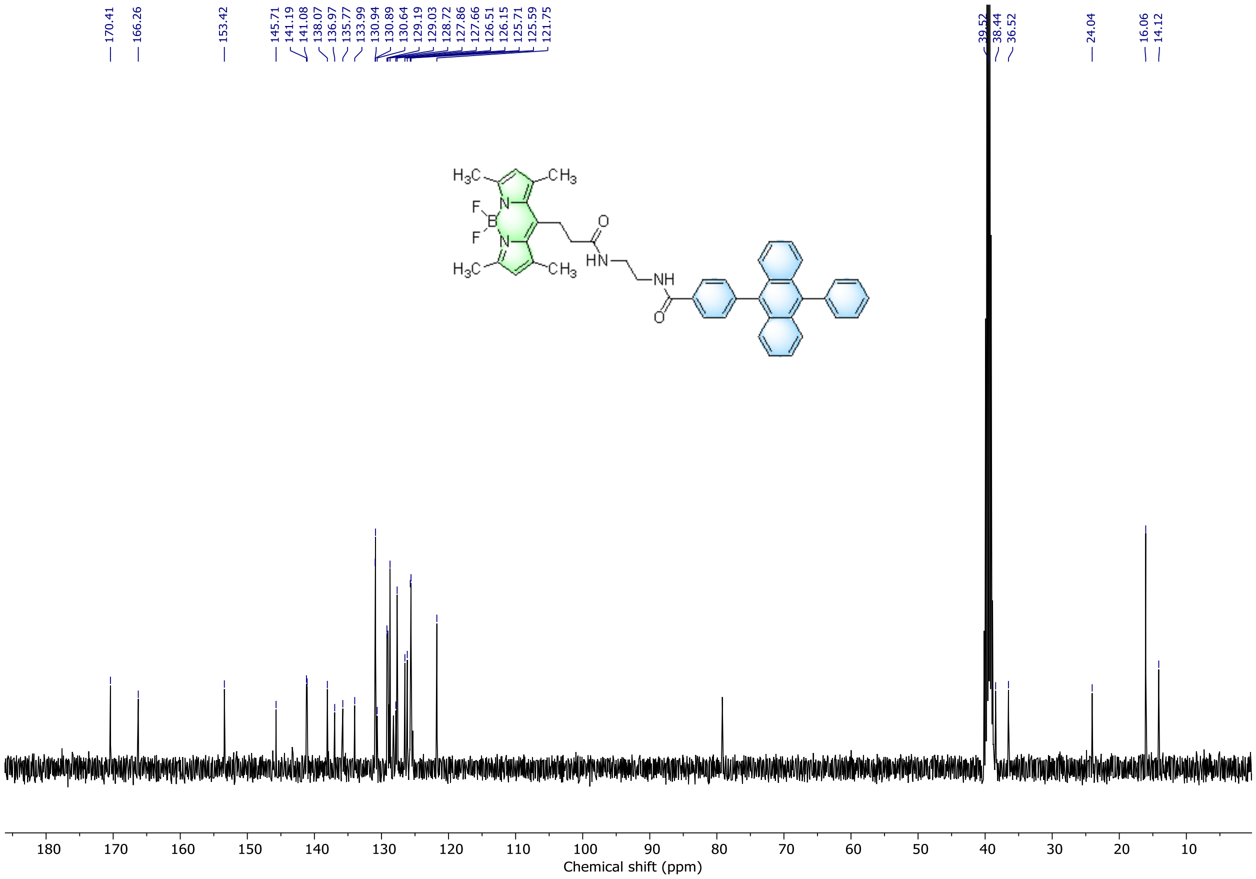

Figure S15. ^{13}C NMR spectrum of **11** in DMSO-d_6.

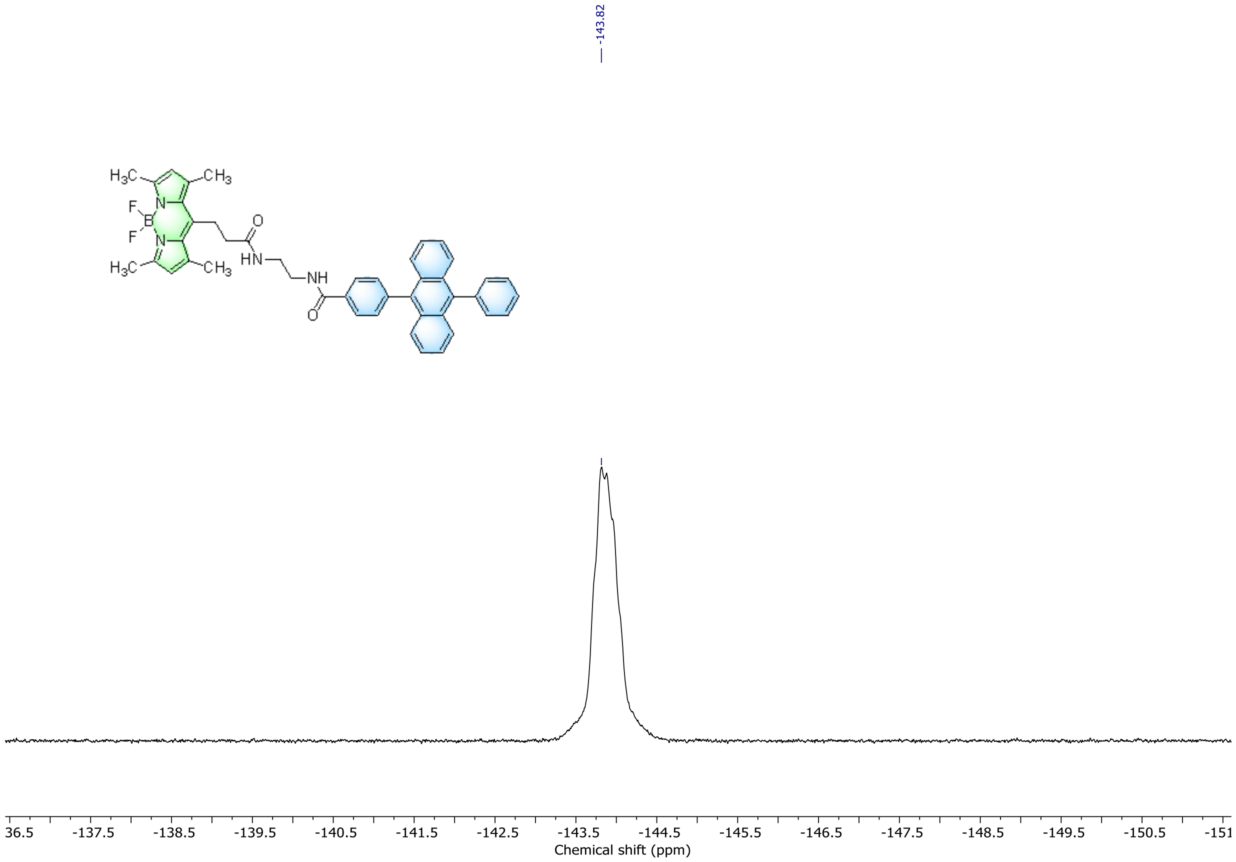

Figure S16. ^{19}F NMR spectrum of **11** in DMSO-d_6.

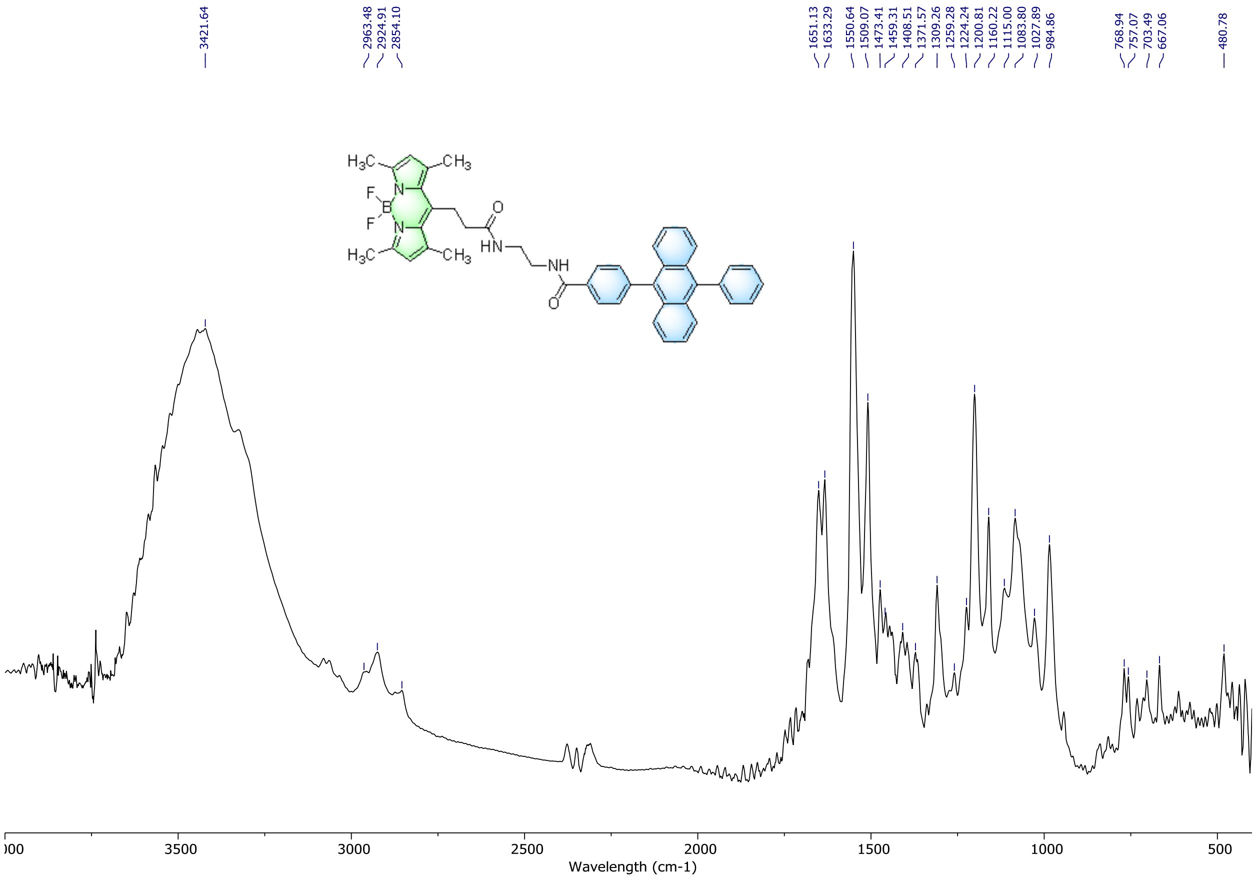

Figure S17. IR spectrum of **11**.

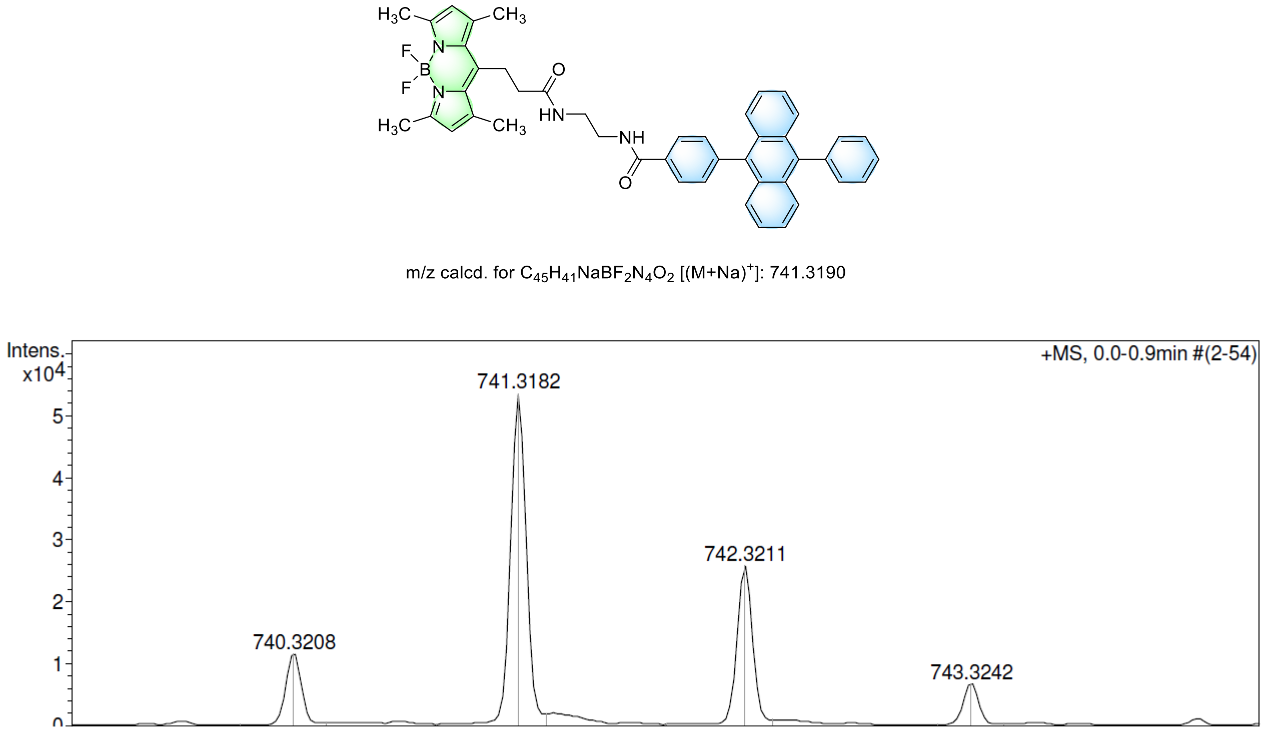

Figure S18. Mass-spectrum of **11**.

4. Absorption and Emission Spectra

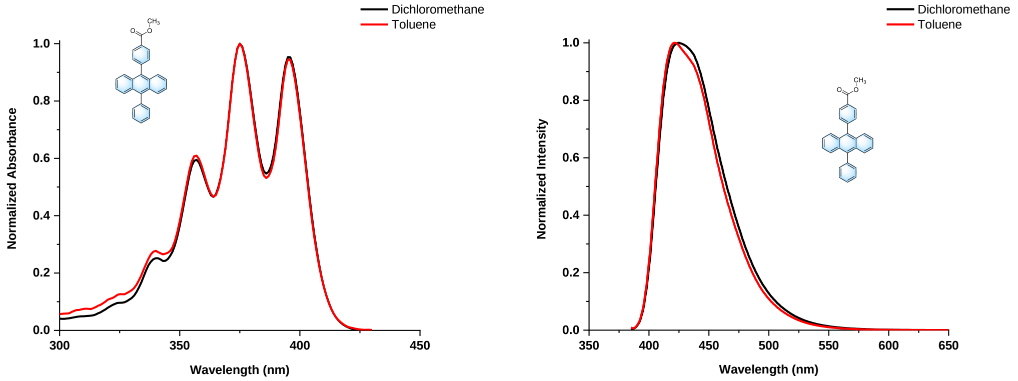

Figure S19. Normalized UV-visible absorption (top) and emission (bottom) spectra of **DPA-COOMe 6** in dichloromethane and toluene at room temperature. Excitation at 375 nm.

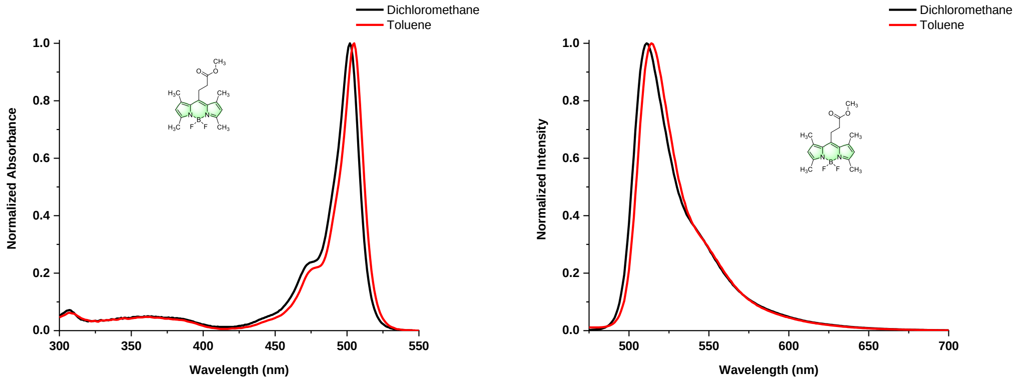

Figure S20. Normalized UV-visible absorption (top) and emission (bottom) spectra of **BODIPY-COOMe 8** in dichloromethane and toluene at room temperature. Excitation at 475 nm.

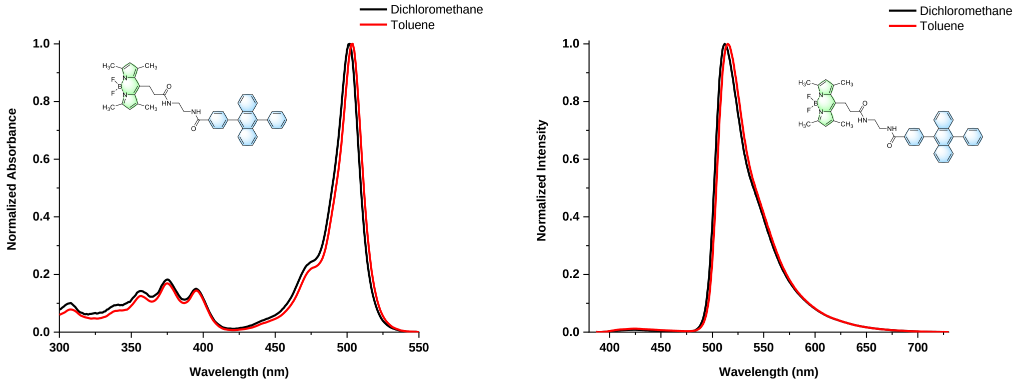

Figure S21. Normalized UV-visible absorption (top) and emission (bottom) spectra of **BODIPY-DPA 11** in dichloromethane and toluene at room temperature. Excitation at 375 nm.

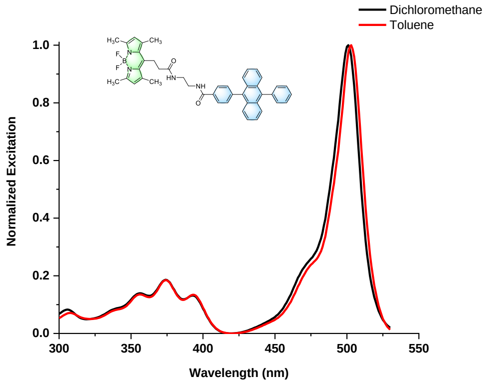

Figure S22. Normalized fluorescence excitation spectra of **BODIPY-DPA 11** in dichloromethane and toluene at room temperature. Emission at 520 nm.

5. References

1. West, M.A. Developments in Instrumentation and Techniques. In *Photochemistry*; Bryce-Smith, D., Ed.; Photochemistry; Royal Society of Chemistry: Cambridge, 1979; Vol. 10, pp. 3–116 ISBN 978-0-85186-590-4.
2. O'Connor, D.; Phillips, D. *Time-Correlated Single Photon Counting*; Elsevier: London, 1984; ISBN 9780125241403.
3. Eaton, D.F. Recommended Methods for Fluorescence Decay Analysis. *Pure Appl. Chem.* **1990**, *62*, 1631–1648, doi:10.1351/pac199062081631.
4. Valeur, B.; Berberan-Santos, M.N. *Molecular Fluorescence: Principles and Applications*; 1st ed.; Wiley, 2012; ISBN 978-3-527-32837-6, pp. 213–261.
5. Ye, Q.; Zheng, F.; Zhang, E.; Bisoyi, H.K.; Zheng, S.; Zhu, D.; Lu, Q.; Zhang, H.; Li, Q. Solvent Polarity Driven Helicity Inversion and Circularly Polarized Luminescence in Chiral Aggregation Induced Emission Fluorophores. *Chem. Sci.* **2020**, *11*, 9989–9993, doi:10.1039/D0SC04179C.
6. Pakhomov, A.A.; Kononevich, Y.N.; Stukalova, M. V.; Svidchenko, E.A.; Surin, N.M.; Cherkaev, G. V.; Shchegolikhina, O.I.; Martynov, V.I.; Muzafarov, A.M. Synthesis and Photophysical Properties of a New BODIPY-Based Siloxane Dye. *Tetrahedron Lett.* **2016**, *57*, 979–982, doi:10.1016/j.tetlet.2016.01.059.
